# TLR3 and GLUL orchestrate inflammatory and homeostatic imbalance in osteoarthritis

**DOI:** 10.3389/fimmu.2025.1650375

**Published:** 2026-01-23

**Authors:** Jing Wang, Shenghao Xu, Bo Chen, Peiqiang Peng, Kai Wang, Yanguo Qin

**Affiliations:** 1 Department of Orthopedics, The Second Hospital of Jilin University, Changchun, Jilin, China; 2 Joint International Research Laboratory of Ageing Active Strategy and Bionic Health in Northeast Asia of Ministry of Education, Jilin University, Changchun, Jilin, China; 3 Department of Medical Rehabilitation, School of Nursing, Jilin University, Changchun, China

**Keywords:** cell death, GLUL, immune infiltration, inflammation, osteoarthritis, TLR3

## Abstract

**Introduction:**

Osteoarthritis (OA) is a degenerative joint disease marked by chronic inflammation, extracellular matrix degradation, and dysregulated cell death. The roles of apoptosis-, autophagy-, and ferroptosis-related genes in OA pathogenesis remain unclear.

**Methods:**

Integrated bioinformatics analyses were conducted on public GEO datasets to identify apoptosis-autophagy-ferroptosis-related genes (AAFRGs). TLR3 and GLUL were identified using LASSO, random forest, and SVM-RFE algorithms. Immune infiltration analysis, immunohistochemistry, and functional assays in human chondrocytes were performed, and ACLT-induced rat OA models were used for *in vivo* validation.

**Results:**

TLR3 was upregulated and associated with pro-inflammatory immune cells, while GLUL was downregulated and correlated with anti-inflammatory signatures. TLR3 knockdown reduced inflammation, apoptosis, and aberrant mineralization, partially restoring extracellular matrix integrity. GLUL overexpression promoted cellular homeostasis. In rats, TLR3 inhibition and PRP treatment decreased pro-inflammatory cytokines (IL-1β, TNF-α), reduced matrix-degrading enzymes (MMP3, MMP13), and restored GLUL and IL-10 levels.

**Discussion/Conclusion:**

TLR3 and GLUL orchestrate inflammatory responses and homeostatic imbalance in OA, representing potential biomarkers and therapeutic targets for diagnosis and intervention.

## 1 Introduction

Osteoarthritis (OA) is a highly prevalent degenerative joint disease characterized by chronic joint pain, swelling, deformities, and impaired mobility, significantly reducing patients’ quality of life and imposing substantial physical, psychological, and economic burdens worldwide (1, 2). Current treatments for OA are largely limited to symptom relief through analgesics, anti-inflammatory drugs, and ultimately joint replacement surgery; however, these approaches do not address the underlying pathological mechanisms or halt disease progression, underscoring the urgent need to identify novel therapeutic targets (3–5).

The pathogenesis of OA is complex and multifactorial, involving mechanical stress, genetic susceptibility, metabolic dysregulation, and chronic low-grade inflammation within the joint microenvironment (6, 7). Progressive degeneration of articular cartilage is a hallmark of OA, often preceded or accompanied by changes in the subchondral bone microenvironment and synovial inflammation (8, 9). Aberrant mechanical loading induces microfractures at the subchondral bone and osteochondral junction, triggering bone remodeling processes that contribute to subchondral sclerosis and osteophyte formation, thereby exacerbating joint deformity and cartilage degradation. Additionally, synovial inflammation characterized by synovial hyperplasia, fibrosis, and increased vascularization leads to the secretion of pro-inflammatory cytokines and metabolic factors by synovial fibroblasts and macrophages, which in turn disrupt chondrocyte homeostasis, causing hypertrophy, fibrosis, apoptosis, and extracellular matrix breakdown (10).

Regulated cell death mechanisms—namely autophagy, apoptosis, and ferroptosis—are essential for maintaining tissue homeostasis and modulating inflammatory responses (11), While their dysregulation has been well characterized in diseases such as cancer and neurodegeneration (12), where they influence immune responses and disease outcomes, their specific roles in osteoarthritis (OA) are only beginning to be elucidated. Recent studies have revealed that ferroptosis may serve as a mechanistic link between mechanical stress and OA by promoting chondrocyte death and cartilage damage (13), Meanwhile, impaired autophagy in fibroblast-like synoviocytes (FLSs) has been shown to drive cellular senescence and the senescence-associated secretory phenotype (SASP), further accelerating joint degeneration. Notably, targeting METTL3-mediated m6A RNA modification can restore autophagy and alleviate OA progression in experimental models (14). These findings suggest that distinct cell death pathways contribute to OA through different cellular mechanisms. Identifying key genes regulating these pathways may uncover novel biomarkers and therapeutic targets for OA.

In this study, we applied integrated bioinformatics analyses on multiple GEO transcriptomic datasets to identify apoptosis, autophagy and ferroptosis-associated genes (AAFRGs) differentially expressed in OA. Through machine learning and functional enrichment approaches, we pinpointed two key genes, Toll-like receptor 3 (TLR3) and glutamine synthetase (GLUL), as critical modulators of OA pathogenesis. Notably, TLR3—a toll-like receptor involved in innate immune signaling—was found to be significantly upregulated in OA tissues, promoting inflammatory responses, matrix degradation, and aberrant chondrocyte mineralization. In contrast, GLUL, which regulates glutamine metabolism and redox balance, was downregulated and appeared to exert protective effects by modulating autophagy and ferroptosis pathways. These expression patterns were further validated in clinical cartilage and synovial specimens from OA patients. Functional studies demonstrated that knockdown of TLR3 ameliorates inflammatory and degenerative processes in chondrocytes, while platelet-rich plasma (PRP) treatment effectively suppresses TLR3 and restores GLUL expression *in vivo*. Collectively, these findings reveal a novel immune-cell death axis mediated by GLUL and TLR3, offering promising diagnostic biomarkers and therapeutic targets for OA.

## 2 Materials and methods

### 2.1 Cell culture

Human chondrocyte cell lines C28/I2 and HC-a, and the mouse chondrogenic progenitor cell line ATDC-5 were purchased from Shanghai Aisair Biotechnology Co., Ltd. All cell lines were cultured in Dulbecco’s Modified Eagle Medium (DMEM) supplemented with 10% fetal bovine serum (FBS), 1% penicillin, and 1% streptomycin. Cells were maintained at 37 °C in a humidified atmosphere containing 5% CO_2_, with medium changes every 48 hours. Cells were passaged using trypsin-EDTA digestion when reaching 80–90% confluence under sterile conditions.

### 2.2 Lentiviral vector construction and cell transduction

Lentiviral vectors for overexpression and knockdown were obtained from GeneMax Biotechnology (Shanghai, China). The constructs included:

1. Human GLUL overexpression: PGMLV-CMV-H_GLUL-EF1-ZsGreen1-T2A-Puro.
2. Mouse Glul overexpression: PGMLV-CMV-Mouse_Glul-EF1-ZsGreen1-T2A-Puro.
3. Human TLR3 knockdown: H_TLR3-shRNA1610, H_TLR3-shRNA527, H_TLR3-shRNA708 (PGMLV-ZsGreen1-Puro).
4. Mouse Tlr3 knockdown: Mouse_Tlr3-shRNA580, Mouse_Tlr3-shRNA1256, Mouse_Tlr3-shRNA1424 (PGMLV-ZsGreen1-Puro).

Sequences for shRNAs targeting TLR3 and corresponding negative controls (NC) were as follows:

Human *TLR3* shRNAs:
  NC: 5’-TTCTCCGAACGTGTCACGT-3’.
  shRNA1610: 5’-CCTCTTCGTAACTTGACCATT-3’.
  shRNA527: 5’-TTTGTCAAGCAGAAGAATTTA-3’.
  shRNA708: 5’-AGTTGTCATCGAATCAAATTA-3’.
Mouse *Tlr3* shRNAs:
  Negative control (Scramble): 5’-TTCTCCGAACGTGTCACGT-3’.
  shRNA580: 5’-TATCTTGGATGCAGGATTTAA-3’.
  shRNA1256: 5’-TCTCTGGAGTACAACAATATA-3’.
  shRNA1424: 5’-CTCAACATGGATGACAATAAT-3’.

For transduction, cells were seeded at a density of 3 × 10^5^ cells per well in 6-well plates and cultured for 24 hours. When cell confluence reached 60–70%, lentiviral particles were added at a multiplicity of infection (MOI) of 50 along with 5 μg/mL polybrene. After 48 hours, the medium was replaced. Cells transduced with puromycin resistance vectors were selected with 2 μg/mL puromycin for one week before downstream assays.

### 2.3 Colony formation assay

To assess cell proliferation and clonogenic potential, 1,000 cells per well were seeded in 6-well plates and cultured for 10 days, with medium replaced every 3 days. Colonies were fixed with 4% paraformaldehyde for 15 minutes at room temperature, washed with PBS, and stained with 0.1% crystal violet for 15 minutes. Excess stain was rinsed off with water, and colonies were air-dried before counting.

### 2.4 Cell viability assay (CCK-8)

Cell proliferation was evaluated using the Cell Counting Kit-8 (CCK-8, Dojindo). Cells were seeded in 96-well plates and incubated for 24 hours before treatment. Following exposure to test compounds for 24, 48, or 72 hours, 10 μL of CCK-8 reagent was added per well and incubated for 1.5 hours. Absorbance was measured at 450 nm using a microplate reader. Results were expressed as relative optical density values compared to controls.

### 2.5 Alcian blue staining

Alcian blue staining was performed to detect acidic mucopolysaccharides. Tissue sections or cell cultures were prepared with standard paraffin embedding and deparaffinization or cryosections. Sections were incubated in 1–3% Alcian blue solution (pH 2.5) for 30–60 minutes, rinsed with distilled water or PBS, and dehydrated through graded alcohols (50%, 70%, 95%, 100%). Following dehydration, sections were cleared in xylene and mounted with neutral resin for microscopic examination. Acidic mucopolysaccharides stained blue.

### 2.6 Alizarin red staining

Alizarin Red S staining was used to detect calcium deposits. Sections or cell cultures were incubated with 0.5% Alizarin Red solution (pH 4.2) for 5–10 minutes, rinsed with distilled water or PBS, dehydrated through graded alcohols (70%, 95%, 100%), cleared in xylene, and mounted. Calcium deposits appeared as red to orange-red areas under microscopy.

### 2.7 Western blot

Cells were lysed on ice using RIPA buffer containing PMSF. Lysates were centrifuged to remove debris, and total protein concentration was determined using the BCA assay kit. Protein samples were mixed with loading buffer and separated by SDS-PAGE (80 V for 10 minutes, then 130 V for 60 minutes). Proteins were transferred onto PVDF membranes using a 300 mA transfer system for 90 minutes. Membranes were blocked with 5% non-fat milk or BSA for 1 hour at room temperature, washed with TBST, and incubated with primary antibodies overnight at 4 °C. After washing, membranes were incubated with HRP-conjugated secondary antibodies for 1 hour at room temperature, washed again, and visualized with enhanced chemiluminescence reagents (Servicebio, China). Band intensities were normalized to β-actin. Primary antibodies used included: β-actin (1:10,000, Proteintech), IL-1β (1:2,000, Affinity), TNF-α (1:2,000, Affinity), IL-10 (1:2,000, Affinity).

### 2.8 Enzyme-linked immunosorbent assay

Levels of IL-1β, TNF-α, and IL-10 in cell culture supernatants were measured by ELISA kits according to the manufacturers’ protocols. Briefly, microplate wells coated with specific capture antibodies were incubated with samples for 1–2 hours, washed, then incubated with enzyme-linked detection antibodies. After washing, substrate solution was added, and color development was measured spectrophotometrically at the appropriate wavelength. Concentrations were calculated based on standard curves.

### 2.9 TUNEL assay

Apoptotic DNA fragmentation was detected using the Terminal deoxynucleotidyl transferase dUTP nick end labeling (TUNEL) assay. Cells cultured on coverslips were fixed with 4% paraformaldehyde for 15–30 minutes, washed with PBS, and permeabilized with a suitable permeabilization buffer for 5–10 minutes. TUNEL reaction mixture was added and incubated for 1–2 hours at 37 °C. After washing, nuclei were counterstained with DAPI. Fluorescence microscopy was used to detect apoptotic cells, indicated by red fluorescence at DNA break sites.

### 2.10 Immunohistochemistry

Tissue samples were fixed in 4% paraformaldehyde, embedded in paraffin, and sectioned at 4 μm thickness. After deparaffinization and antigen retrieval in citrate buffer (pH 6.0), sections were blocked with 5% normal serum and incubated overnight at 4 °C with primary antibodies against target proteins including glutamine synthetase (GLUL), toll-like receptor 3 (TLR3), and IL-1β (dilution 1:100). Following washes, sections were incubated with HRP-conjugated secondary antibodies at room temperature for 1 hour. Immunoreactivity was visualized using DAB substrate and counterstained with hematoxylin. After dehydration and mounting, slides were examined under light microscopy. Five random high-power fields per section were semi-quantitatively scored based on staining intensity and percentage of positive cells to generate an overall expression score.

### 2.11 Animal experiments

All animal procedures were approved by the Institutional Animal Care and Use Committee (IACUC) of The Second Hospital of Jilin University. Thirty adult male Sprague-Dawley (SD) rats (weight: ~200 g) were purchased from Beijing Vital River Laboratory Animal Technology Co., Ltd. Rats were randomly assigned to four groups using a computer-generated randomization sequence: sham-operated control (n=8), OA model (n=8), TLR3 inhibitor treatment group (n=7), and PRP treatment group (n=7).

Osteoarthritis (OA) was induced by anterior cruciate ligament transection (ACLT) of the right knee under sodium pentobarbital anesthesia (40–60 mg/kg, intraperitoneally). Sham-operated rats underwent arthrotomy without ligament transection. Postoperative care included daily monitoring and intraperitoneal injection of penicillin (20,000–40,000 IU/kg) for 3 days to prevent infection. No mortality or severe complications occurred during the study; all animals survived until the experimental endpoint.

Starting at week 4 post-surgery, intra-articular injections were administered only to the operated right knee three times per week for 4 weeks under sterile conditions. The TLR3 inhibitor group received 50 μL of CU-CPT 4a (10 μM in saline). The PRP group received 50 μL of platelet-rich plasma. Control and model groups were injected with equal volumes of saline.

Knee joints were harvested at week 8 via CO_2_ inhalation. Histological and functional assessments were performed blinded to group assignment. OA severity was assessed based on joint circumference, stride length, step frequency, and range of motion using standardized measurement protocols. HE and toluidine blue staining were used for cartilage evaluation. OARSI scoring was conducted only on the operated right knee.

These *in vivo* experiments comprehensively demonstrate the utility of ACLT-induced OA rats for studying TLR3/GLUL signaling and provide a basis for further investigation of TLR3-targeted therapeutic strategies in OA.

### 2.12 Hematoxylin and eosin staining

Knee joint specimens were harvested at 8 weeks post-surgery and fixed in 4% paraformaldehyde for 48 hours at room temperature. Following decalcification in 10% EDTA solution for 4 weeks (changed every 3 days), tissues were dehydrated, embedded in paraffin, and sectioned at 5 μm thickness. Sections were stained with hematoxylin for 5 minutes and eosin for 3 minutes. After dehydration and mounting, stained sections were examined under a light microscope to evaluate cartilage surface integrity, chondrocyte distribution, synovial hyperplasia, and subchondral bone structure.

### 2.13 Toluidine blue staining

For proteoglycan assessment, adjacent paraffin sections (5 μm) were stained with 0.1% toluidine blue in 0.1 M acetate buffer (pH 4.0) for 5–10 minutes at room temperature. Sections were rinsed with distilled water, dehydrated, cleared in xylene, and mounted with neutral resin. Toluidine blue-positive areas indicated the presence of sulfated glycosaminoglycans in cartilage. Staining intensity and distribution were evaluated to assess cartilage degeneration and proteoglycan loss.

### 2.14 Clinical tissue sample collection

This study was approved by the Ethics Committee of the Second Hospital of Jilin University (Approval No. 2024-159). Knee cartilage and synovial tissue samples were collected from OA patients undergoing knee replacement surgery at the Orthopedics Department between January 30 and April 20, 2024. Normal periarticular tissues from patients without OA served as controls. Inclusion criteria were diagnosis of knee OA, age over 18 years, voluntary participation, and signed informed consent in accordance with the Helsinki Declaration.

### 2.15 Data acquisition and preprocessing

The Gene Expression Omnibus (GEO) database (http://www.ncbi.nlm.nih.gov/geo) was searched using the keyword “osteoarthritis” to obtain relevant transcriptomic datasets. Four datasets were selected based on sample size and data quality: GSE51588 [16] (10 healthy controls, 40 OA patients), GSE117999 [17] (12 healthy controls, 12 OA patients), GSE55235 [18] (10 healthy controls, 20 OA patients), and GSE82107 [19] (7 healthy controls, 10 OA patients). GSE51588 (GPL13497) and GSE117999 (GPL20844), both generated on Agilent microarray platforms, were used as training cohorts for model construction, whereas GSE55235 (GPL96) and GSE82107 (GPL570), generated on Affymetrix microarray platforms, served as independent validation cohorts. To ensure comparability across datasets generated from different microarray platforms, all expression data were preprocessed in R using platform-specific normalization procedures. Specifically, the Agilent-based training datasets were normalized using the limma package, while the Affymetrix-based validation datasets were normalized using the Robust Multi-array Average (RMA) algorithm implemented in the affy package. Following normalization, probe sets were mapped to official gene symbols according to the corresponding platform annotation files. When multiple probes mapped to the same gene, the median (or mean) expression value was used to represent gene-level expression. Expression matrices were then merged at the gene symbol level by retaining the intersecting genes across datasets. To mitigate systematic non-biological variations introduced by different platforms and studies, batch effects were adjusted using the ComBat algorithm implemented in the sva package. Cross-dataset validation was subsequently performed to evaluate the robustness and generalizability of the constructed model. Since all datasets were obtained from publicly available databases, no additional ethical approval or informed consent was required.

### 2.16 Identification of apoptosis-autophagy-ferroptosis-related genes and differential expression analysis

Gene sets related to apoptosis were obtained from the Kyoto Encyclopedia of Genes and Genomes (KEGG) database (Apoptosis-related genes were retrieved from the KEGG pathway hsa04210) [20], autophagy-related genes from the autophagy database (http://www.autophagy.lu/), and ferroptosis-related genes from FerrDb (http://www.zhounan.org/ferrdb/current/).

These collectively defined apoptosis-autophagy-ferroptosis-related genes (AAFRGs) were subjected to differential expression analysis using the “limma” R package. Genes with significant differential expression between OA and control samples were identified based on adjusted p-values (<0.05).

### 2.17 Functional enrichment analysis

Gene Ontology (GO) and KEGG pathway enrichment analyses were performed to explore the biological functions of the differentially expressed AAFRGs. The analyses utilized the “clusterProfiler,” “ggplot2,” “org.Hs.eg.db,” and “enrichplot” R packages. Enriched pathways and terms with adjusted p-values < 0.05 were considered statistically significant.

### 2.18 Screening of key osteoarthritis-related genes

Three machine learning algorithms were applied to prioritize characteristic NAFRGs: Least Absolute Shrinkage and Selection Operator (LASSO), Support Vector Machine Recursive Feature Elimination (SVM-RFE), and Random Forest (RF). The “glmnet” package performed 10-fold cross-validation for LASSO to identify relevant features. SVM-RFE analysis was conducted using the “e1071” and “svmRadial” packages. The RF model was constructed using the “randomForest” package, ranking genes by the Gini importance index. Genes intersecting across all three methods were considered key OA biomarkers. The predictive performance of these genes was evaluated via Receiver Operating Characteristic (ROC) curve analysis using the “pROC” package, with the area under the curve (AUC) indicating diagnostic accuracy.

### 2.19 Gene set enrichment analysis and gene set variation analysis

GSEA was performed using the “c2.cp.kegg.v11.0” gene set from the Molecular Signatures Database (MSigDB, http://software.broadinstitute.org/gsea/msigdb) to investigate the biological pathways associated with key genes. The significance threshold was set at adjusted p-value < 0.05 with 1000 permutations to normalize enrichment scores.

To explore the variation in pathway activity across samples, Gene Set Variation Analysis (GSVA) was performed using the GSVA R package (v1.46.0). The “h.all.v7.5.symbols.gmt” reference gene set from the MSigDB database was applied, with the following parameters: method = “gsva”, min.sz = 10, max.sz = 500, and mx.diff = TRUE. Enrichment scores were normalized per sample, and pathways with adjusted p < 0.05 were considered statistically significant.

### 2.20 Immune cell infiltration analysis

The CIBERSORT algorithm (http://cibersortx.stanford.edu) was applied to quantify the relative proportions of 22 immune cell types in OA and control samples based on gene expression profiles. Spearman correlation analysis assessed relationships between key gene expression and immune cell infiltration levels. Visualization was performed using the “ggplot2” R package.

### 2.21 Validation of key genes expression

Expression levels of key genes were validated in independent datasets (GSE55235, GSE82107) using the “ggpubr” and “ggplot2” packages. Statistical significance was determined with adjusted p-values < 0.05.

### 2.22 Statistical analysis

All statistical analyses were performed using R software (version 4.2.0). Continuous variables are presented as mean ± standard deviation (SD) or median with interquartile range (IQR) depending on data distribution. Group comparisons were conducted using Student’s t-test or Mann-Whitney U test as appropriate. Multiple group comparisons were performed using one-way ANOVA or Kruskal-Wallis test followed by *post hoc* analysis. Correlations between gene expression and immune cell infiltration were evaluated using Spearman’s rank correlation coefficient. P-values were adjusted for multiple testing using the Benjamini-Hochberg method, and an adjusted p-value < 0.05 was considered statistically significant. Receiver operating characteristic (ROC) curves and area under the curve (AUC) were calculated to assess the diagnostic performance of candidate genes. Graphical visualization was generated with the “ggplot2” and “pROC” R packages.

## 3 Results

### 3.1 Identification of differentially expressed NAFRGs in osteoarthritis

**Figure 1:**
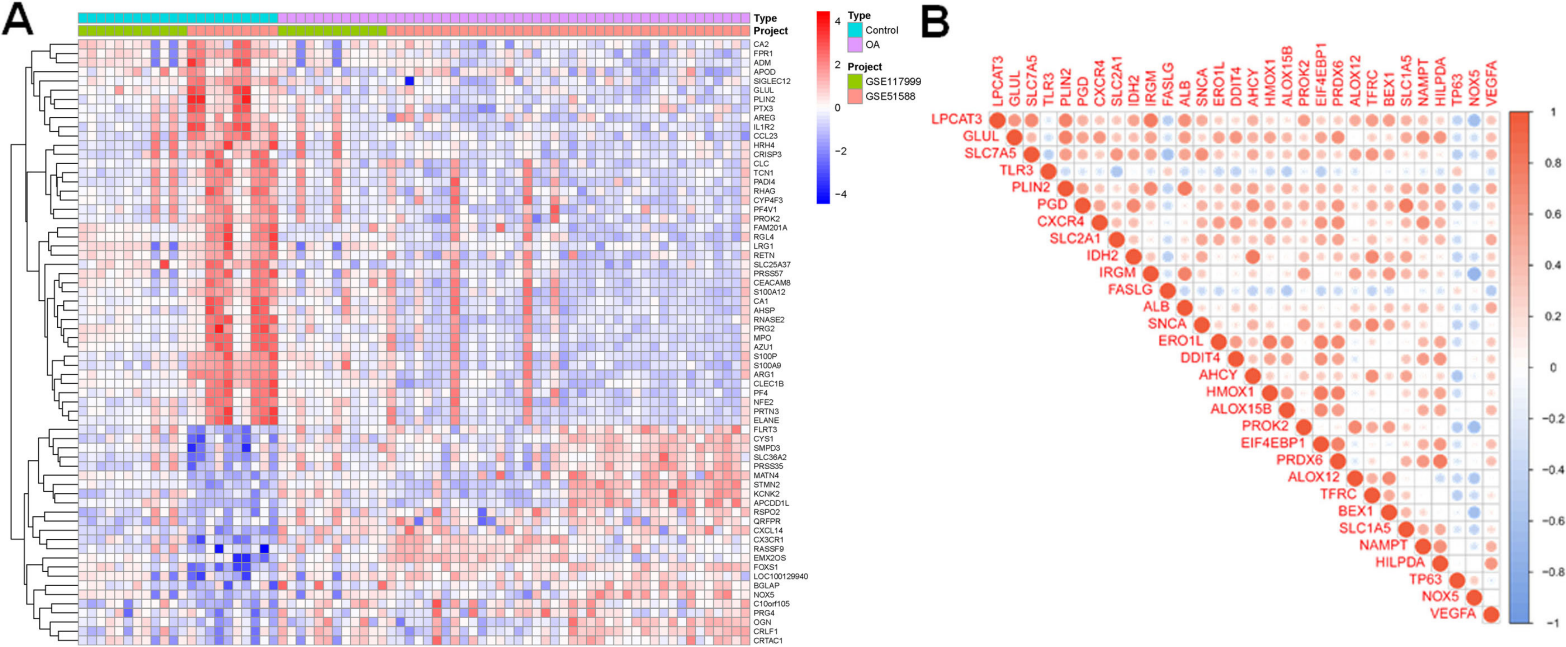
Expression profiling and correlation analysis of NAFRGs in osteoarthritis. **(A)** Heatmap showing differentially expressed genes between OA and healthy control samples in the test datasets (GSE51588 and GSE117999). **(B)** Correlation matrix of differentially expressed NAFRGs, indicating potential gene–gene interactions relevant to OA pathogenesis.

To ensure data reliability and comparability, we selected the publicly available GEO datasets GSE51588 and GSE117999 as the test sets for differential gene expression analysis. Both datasets contain synovial and cartilage tissue samples from patients with OA and healthy controls, generated using comparable microarray platforms with clear clinical annotation and adequate sample sizes. Specifically, GSE51588 includes 40 OA samples and 10 controls, and GSE117999 includes 12 OA and 12 controls, totaling 52 OA and 22 healthy samples. Differential expression analysis identified a total of 66 genes significantly dysregulated in OA samples (**Figure 1A**). By intersecting these with known AAFRGs, we obtained 30 differentially expressed NAFRGs, comprising 13 upregulated and 17 downregulated genes. Correlation analysis revealed that *GLUL* expression was positively associated with *IRGM*, *EIF4EBP1*, *PRDX6*, *HILPDA*, and *TFRC*, and negatively associated with *FASLG*. In contrast, *TLR3* displayed an opposite correlation pattern. These inter-gene relationships are summarized in **Table 1** and visualized in **Figure 1B**, suggesting potential regulatory interactions relevant to OA pathogenesis.

**Table 1:** Correlation between the NAFRGs.

id	logFC	AveExpr	t-statistic	P.Value	adj.P.Val	B-statistic
LPCAT3	-0.65779	2.577456	-6.11751	3.79E-08	2.79E-05	8.507081
GLUL	-1.07302	2.917319	-5.80965	1.37E-07	3.36E-05	7.296721
SLC7A5	-0.87803	2.708964	-5.20804	1.57E-06	0.000232	5.000956
TLR3	0.569912	2.601487	4.994385	3.64E-06	0.000384	4.212285
PLIN2	-1.06787	2.824906	-4.46433	2.74E-05	0.002017	2.329058
PGD	-0.64453	2.863245	-4.31708	4.70E-05	0.002472	1.826796
CXCR4	-0.79477	3.075065	-4.1506	8.55E-05	0.003706	1.271057
SLC2A1	-0.51187	2.935793	-4.1028	0.000101	0.003885	1.113954
IDH2	-0.55735	2.573588	-4.09617	0.000104	0.003885	1.092245
IRGM	-0.75012	2.310517	-4.07159	0.000113	0.003932	1.012008
FASLG	0.613638	2.861177	3.889271	0.000213	0.005443	0.426381
ALB	-0.96149	2.600271	-3.88805	0.000214	0.005443	0.422521
SNCA	-0.60506	2.721212	-3.77447	0.000315	0.007746	0.06669
ERO1L	-0.51366	2.873626	-3.69915	0.000406	0.00935	-0.16537
DDIT4	-0.82565	3.222655	-3.65774	0.000466	0.00966	-0.29158
AHCY	-0.51257	2.624708	-3.64202	0.000491	0.00966	-0.33924
HMOX1	-0.81384	3.343451	-3.63839	0.000497	0.00966	-0.35024
ALOX15B	-0.6298	2.888022	-3.63754	0.000498	0.00966	-0.35279
PROK2	-1.06505	2.459796	-3.56992	0.000622	0.01133	-0.55598
EIF4EBP1	-0.50657	3.003119	-3.5592	0.000644	0.01133	-0.58795
PRDX6	-0.5274	3.266128	-3.51007	0.000756	0.012374	-0.73358
ALOX12	-0.97616	1.783837	-3.48033	0.000832	0.01304	-0.82104
TFRC	-0.54911	2.526148	-3.28711	0.001532	0.020369	-1.37606
BEX1	-0.67687	2.324218	-3.28153	0.001559	0.020369	-1.39176
SLC1A5	-0.51823	2.655181	-3.20279	0.001986	0.023238	-1.6109
NAMPT	-0.62909	3.014606	-3.08687	0.00282	0.028466	-1.9262
HILPDA	-0.83255	3.715442	-3.07136	0.002953	0.029409	-1.9677
TP63	0.603406	2.856938	3.047309	0.003171	0.030755	-2.03176
NOX5	1.134792	4.044012	3.008152	0.00356	0.033209	-2.1352
VEGFA	-0.53033	2.734136	-2.85458	0.005547	0.042697	-2.53071

### 3.2 Functional enrichment analysis of differentially expressed NAFRGs

**Figure 2:**
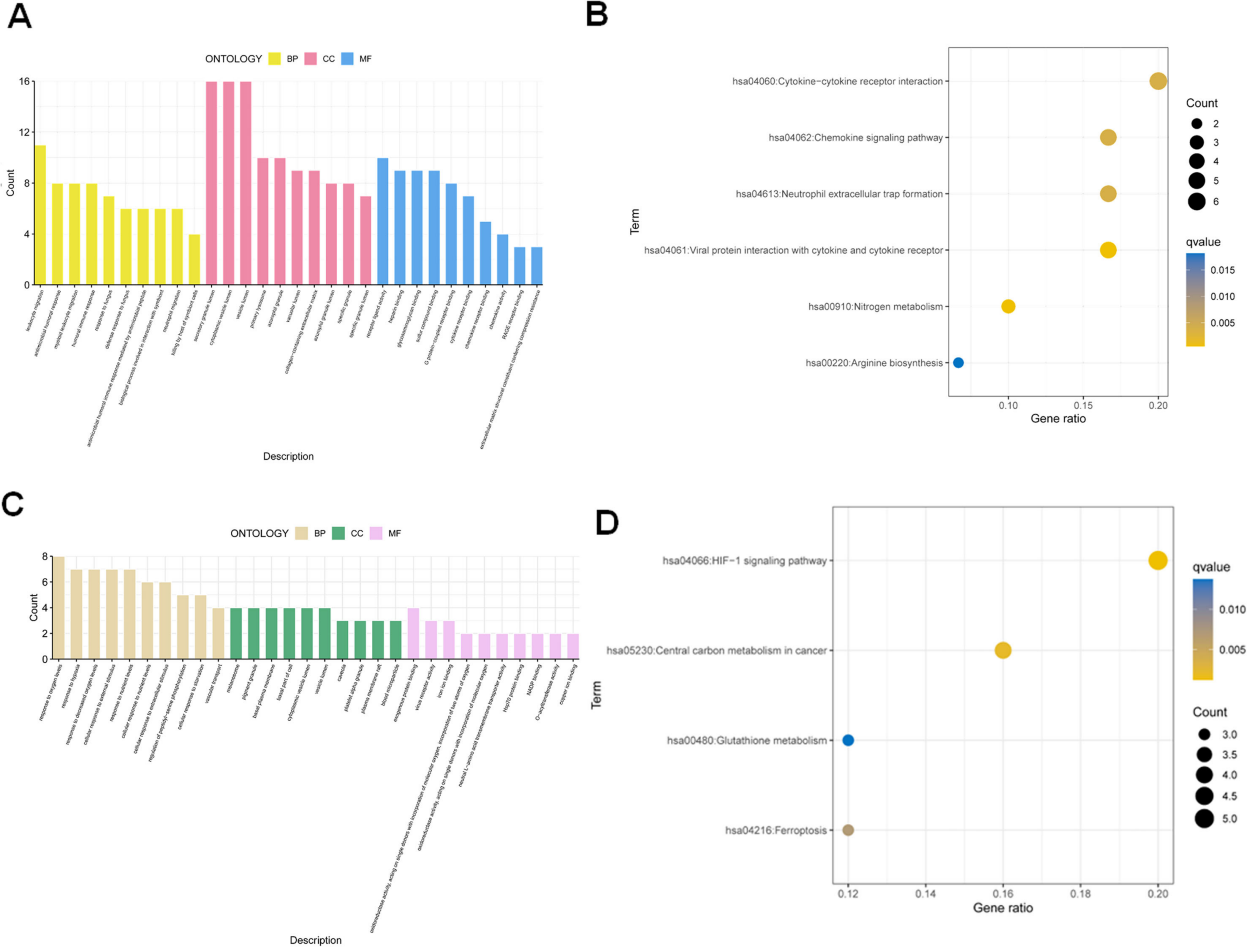
Functional enrichment analysis of differentially expressed genes in OA and NAFRGs. **(A)** Gene Ontology (GO) analysis of OA-related differentially expressed genes. In biological processes (BP), genes were enriched in leukocyte migration, humoral immune response, and neutrophil migration. In cellular components (CC), enrichment was observed in cytoplasmic vesicle lumen, azurophil granule lumen, and related structures. In molecular functions (MF), genes were associated with receptor–ligand activity and cytokine receptor binding. **(B)** KEGG pathway analysis of OA-related genes showing enrichment in cytokine–cytokine receptor interaction, chemokine signaling pathway, neutrophil extracellular trap formation, viral protein interactions with cytokines and cytokine receptors, and nitrogen metabolism. **(C)** GO analysis of differentially expressed NAFRGs. BP terms included cellular response to external stimulus and vascular transport; CC terms included basal plasma membrane and plasma membrane raft; MF terms included iron ion binding and NADP binding. **(D)** KEGG pathway analysis of NAFRGs showing enrichment in the HIF-1 signaling pathway, central carbon metabolism in cancer, glutathione metabolism, and ferroptosis.

To explore the biological significance of NAFRGs in OA, we conducted GO and KEGG enrichment analyses on the 66 OA-related DEGs and the 30 intersecting NAFRGs. GO analysis of OA-related DEGs revealed enrichment in immune-related biological processes such as leukocyte migration and neutrophil activation. These genes were primarily located in vesicle-associated compartments and involved in receptor–ligand activity and cytokine binding (**Figure 2A**). KEGG analysis further indicated enrichment in cytokine–cytokine receptor interaction, chemokine signaling, and NET formation (**Figure 2B**). For the 30 differentially expressed NAFRGs, GO terms were enriched in cellular responses to external stimuli and membrane-associated components such as the plasma membrane raft. Their molecular functions were linked to iron ion binding and NADP binding, implicating ferroptosis and oxidative stress pathways (**Figure 2C**). KEGG analysis showed significant enrichment in the HIF-1 signaling pathway, glutathione metabolism, and ferroptosis (**Figure 2D**), suggesting that NAFRGs may modulate OA progression through metabolic stress and programmed cell death mechanisms.

### 3.3 Identification and validation of key diagnostic NAFRGs in osteoarthritis via integrated machine learning approaches

**Figure 3:**
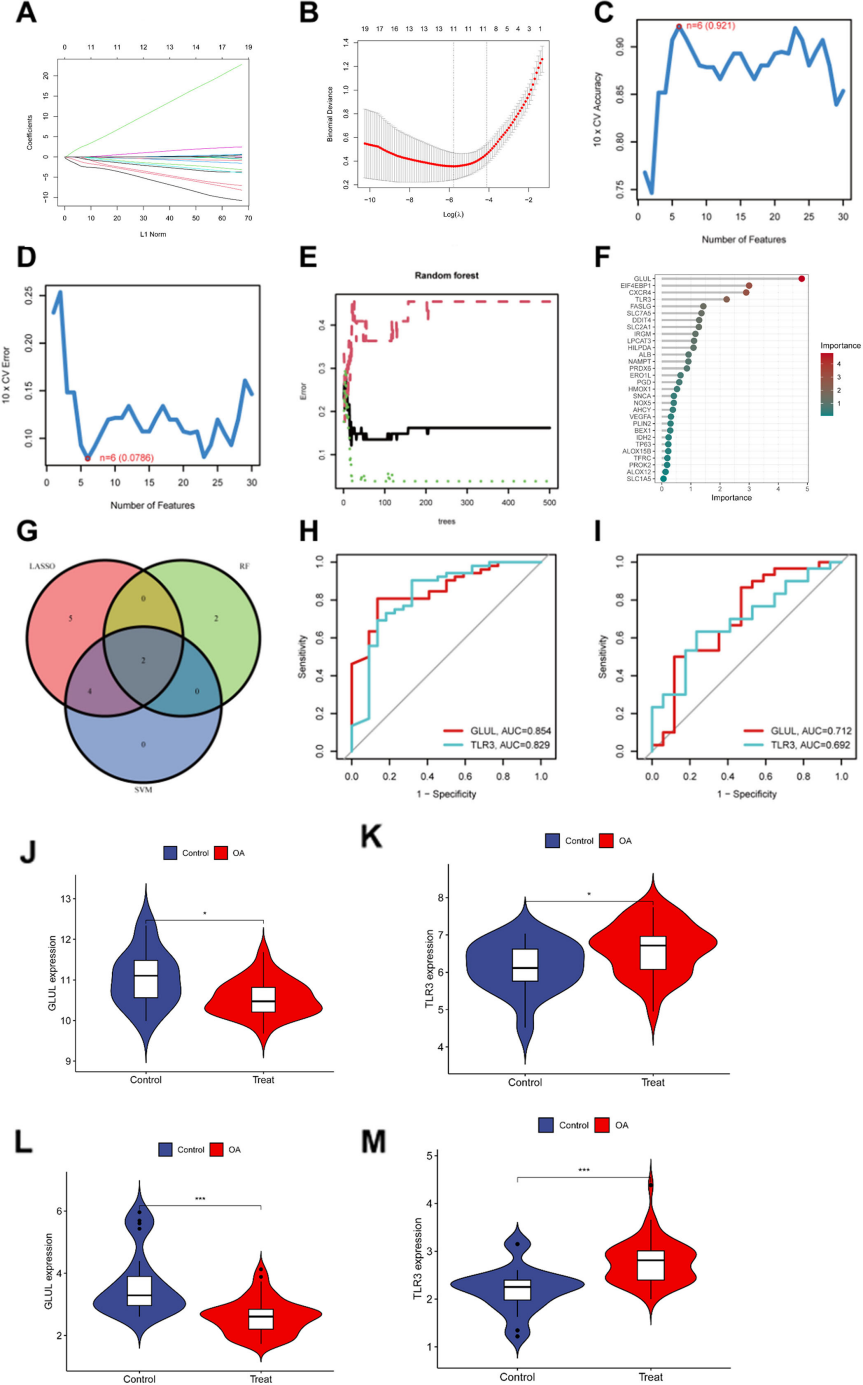
Identification and validation of key NAFRGs associated with osteoarthritis. **(A)** LASSO coefficient profile of necroptosis–autophagy–ferroptosis-related genes (NAFRGs) plotted against the log(λ) sequence, showing the shrinkage of coefficients with increasing penalty. **(B)** Tenfold cross-validation curve for LASSO logistic regression, where the optimal λ value (vertical dashed line) corresponds to the minimum mean cross-validation error. **(C)** Feature selection curve of the Support Vector Machine–Recursive Feature Elimination (SVM-RFE) algorithm, displaying the number of features versus model classification accuracy. **(D)** Root mean square error (RMSE) plot from SVM-RFE analysis, identifying the optimal feature subset with the lowest RMSE. **(E)** Variable importance ranking of NAFRGs derived from the Random Forest algorithm, based on the Mean Decrease Gini index. **(F)** Error rate curve of the Random Forest model showing classification accuracy with an increasing number of decision trees. **(G)** Venn diagram of intersecting genes identified by all three machine learning algorithms (LASSO, SVM-RFE, and Random Forest). **(H)** Receiver operating characteristic (ROC) curves for GLUL and TLR3 in the training datasets (GSE51588 and GSE117999), both showing strong diagnostic performance (AUC > 0.80). **(I)** ROC curves for GLUL and TLR3 in the validation datasets (GSE55235 and GSE82107), confirming predictive ability (AUC > 0.69). **(J, K)** Expression levels of GLUL and TLR3 in the training datasets, comparing osteoarthritis (OA) patients and healthy controls. **(L, M)** Expression levels of GLUL and TLR3 in the independent validation datasets, comparing OA and normal samples.

To identify the most robust NAFRGs with diagnostic value for osteoarthritis, three independent machine learning algorithms—Least Absolute Shrinkage and Selection Operator (LASSO), Support Vector Machine–Recursive Feature Elimination (SVM-RFE), and Random Forest (RF)—were applied to the test datasets (GSE51588 and GSE117999). LASSO regression identified 12 candidate genes with non-zero coefficients, indicating a strong association with OA status (**Figures 3A, B**). The SVM-RFE algorithm achieved optimal classification performance (accuracy = 0.921, RMSE = 0.0786) and selected six key NAFRGs based on feature elimination (**Figures 3C, D**). The RF algorithm further evaluated gene importance by calculating Gini indices, identifying four NAFRGs with an importance score exceeding 0.9 (**Figures 3E, F**). Integrative analysis of the outputs from all three algorithms revealed two consistently overlapping genes, GLUL and TLR3, which were designated as key NAFRGs in OA (**Figure 3G**). To assess their diagnostic performance, Receiver Operating Characteristic (ROC) curves were generated. In the training cohort, both genes demonstrated high discriminatory ability with area under the curve (AUC) values exceeding 0.80 (**Figure 3H**). Validation in independent datasets (GSE55235 and GSE82107) confirmed the diagnostic relevance, with AUC values above 0.69 for both genes (**Figure 3I**). These findings suggest that GLUL and TLR3 may serve as reliable diagnostic biomarkers for distinguishing OA from healthy controls.

Further expression validation of GLUL and TLR3 was performed in both the training and validation datasets. Consistent with the diagnostic findings, GLUL expression was significantly downregulated in OA samples compared to healthy controls, while TLR3 expression was significantly upregulated in OA samples. Specifically, in the training datasets (GSE51588 and GSE117999), GLUL and TLR3 exhibited distinct differential expression patterns (**Figures 3J, K**). These expression patterns were reproduced in the validation datasets (GSE55235 and GSE82107), with similar trends observed for both genes (**Figures 3L, M**). All expression data are presented as boxplots with median, interquartile range, and outliers. Statistical significance was assessed with appropriate tests, and p-values < 0.05 were considered significant.

### 3.4 GSVA and GSEA analysis of key genes in OA reveals distinct metabolic and signaling pathway regulation

**Figure 4:**
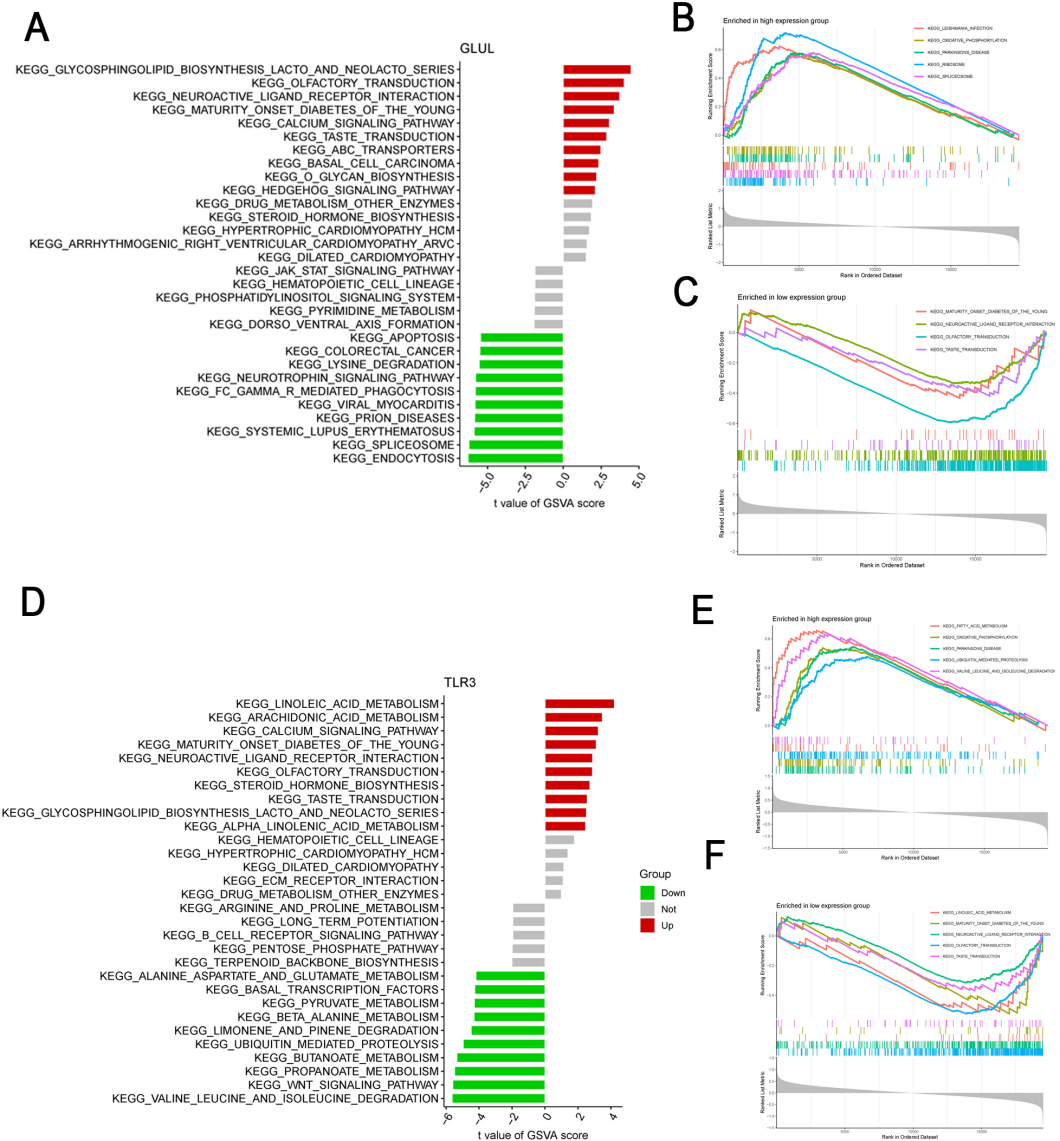
GSVA and GSEA-KEGG pathway analysis of GLUL and TLR3 in osteoarthritis samples. **(A)** GSVA analysis showing variation of pathway activity scores associated with GLUL expression across samples. Each bar represents a KEGG pathway’s enrichment score in relation to GLUL. **(B)** GSEA identifying KEGG pathways positively enriched in samples with high GLUL expression. Enrichment scores reflect the degree of association between GLUL and each pathway. **(C)** GSEA identifying KEGG pathways negatively enriched in samples with high GLUL expression. **(D)** GSVA analysis showing variation of pathway activity scores associated with TLR3 expression across samples. **(E)** GSEA identifying KEGG pathways positively enriched in samples with high TLR3 expression. **(F)** GSEA identifying KEGG pathways negatively enriched in samples with high TLR3 expression.

Given the unclear roles of the key genes in the pathogenesis of OA, we performed Gene Set Variation Analysis (GSVA) and Gene Set Enrichment Analysis (GSEA) to investigate their potential functions and biological significance. GSVA results revealed that GLUL was downregulated in pathways related to apoptosis, Fc gamma R-mediated phagocytosis, and systemic lupus erythematosus, while it was upregulated in basal cell carcinoma and ABC transporter pathways (**Figure 4A**). In contrast, TLR3 showed upregulation in the calcium signaling pathway, neuroactive ligand-receptor interaction, and steroid hormone biosynthesis pathways, but downregulation in propanoate metabolism, WNT signaling pathway, and pyruvate metabolism (**Figure 4D**). Consistent with these findings, GSEA demonstrated that GLUL was significantly enriched and upregulated in oxidative phosphorylation and ribosome pathways (**Figure 4B**), yet downregulated in neuroactive ligand-receptor interactions (**Figure 4C**). Meanwhile, TLR3 was positively enriched in oxidative phosphorylation and ubiquitin-mediated proteolysis pathways (**Figure 4E**), but negatively enriched in neuroactive ligand-receptor interaction, maturity-onset diabetes of the young, and linoleic acid metabolism pathways (**Figure 4F**). Together, these analyses consistently indicate that GLUL and TLR3 are involved in multiple key metabolic and signaling pathways, suggesting their important roles in the molecular mechanisms underlying OA.

### 3.5 Immune cell infiltration patterns related to GLUL and TLR3 in OA

The pathogenesis of OA is multifactorial, with growing evidence implicating the immune microenvironment as a key contributor (15, 16). To further quantify these associations, the transcriptomic data used for immune deconvolution were derived from cartilage and osteochondral tissues, rather than synovial samples. The relative fractions of 22 immune cell types were estimated using CIBERSORT, and Spearman correlation analysis was performed to assess relationships between key gene expression (GLUL and TLR3) and immune cell infiltration. Detailed correlation coefficients (r values and adjusted p-values) are provided in **Tables 2**, **3**.

**Table 2:** Correlation analysis between GLUL expression and immune cell infiltration in cartilage and osteochondral tissues.

Gene	Cell	cor	pvalue
GLUL	B cells naive	-0.020947773	0.934247553
GLUL	B cells memory	0.169631634	0.500996946
GLUL	Plasma cells	-0.045716311	0.857053116
GLUL	T cells CD8	0.096173939	0.704227251
GLUL	T cells CD4 naive	-0.414943341	0.086848522
GLUL	T cells CD4 memory resting	-0.0233741	0.926650011
GLUL	T cells CD4 memory activated	0.421854753	0.081202107
GLUL	T cells follicular helper	0.635988951	0.00455209
GLUL	T cells regulatory (Tregs)	-0.368611304	0.132271962
GLUL	T cells gamma delta	0.263500939	0.290740616
GLUL	NK cells resting	-0.166298065	0.509569816
GLUL	NK cells activated	0.433288155	0.072458385
GLUL	Monocytes	0.114575344	0.650764728
GLUL	Macrophages M0	0.18879922	0.45308051
GLUL	Macrophages M1	0.169334044	0.501759435
GLUL	Macrophages M2	0.394436078	0.105280195
GLUL	Dendritic cells resting	0.105253832	0.677663636
GLUL	Dendritic cells activated	-0.039711516	0.875680688
GLUL	Mast cells resting	-0.378162436	0.121780272
GLUL	Mast cells activated	0.382936521	0.116762326
GLUL	Eosinophils	0.090542256	0.720870457
GLUL	Neutrophils	0.262136973	0.293337358

Transcriptomic data were derived from cartilage and osteochondral samples. The relative proportions of 22 immune cell types were estimated using the CIBERSORT algorithm. Spearman correlation analysis was performed to assess the associations between GLUL expression levels and immune cell fractions. Correlation coefficients (r values) and adjusted p-values are shown.

**Table 3:** Correlation analysis between TLR3 expression and immune cell infiltration in cartilage and osteochondral tissues.

Gene	Cell	cor	pvalue
TLR3	B cells naive	0.054023205	0.831404128
TLR3	B cells memory	0.050397804	0.842579784
TLR3	Plasma cells	0.181802074	0.470295763
TLR3	T cells CD8	-0.402275938	0.0979308
TLR3	T cells CD4 naive	-0.156641111	0.534788878
TLR3	T cells CD4 memory resting	-0.116870502	0.644202625
TLR3	T cells CD4 memory activated	0.048323919	0.848985773
TLR3	T cells follicular helper	0.336091722	0.172690475
TLR3	T cells regulatory (Tregs)	-0.460505998	0.054459535
TLR3	T cells gamma delta	0.001102514	0.996535811
TLR3	NK cells resting	-0.179256356	0.476638831
TLR3	NK cells activated	0.62292063	0.005754674
TLR3	Monocytes	0.163979392	0.515573266
TLR3	Macrophages M0	0.11929621	0.637294376
TLR3	Macrophages M1	-0.27258651	0.273800952
TLR3	Macrophages M2	0.426331179	0.077691395
TLR3	Dendritic cells resting	-0.194560114	0.439151989
TLR3	Dendritic cells activated	0.268449848	0.281436425
TLR3	Mast cells resting	-0.06284332	0.804347354
TLR3	Mast cells activated	0.248115675	0.320840893
TLR3	Eosinophils	0.252565241	0.311953349
TLR3	Neutrophils	0.274889583	0.269605874

Transcriptomic data from cartilage and osteochondral tissues were used for immune deconvolution. Immune cell fractions were estimated using the CIBERSORT algorithm, followed by Spearman correlation analysis to evaluate the relationships between TLR3 expression and immune cell infiltration.

**Figure 5:**
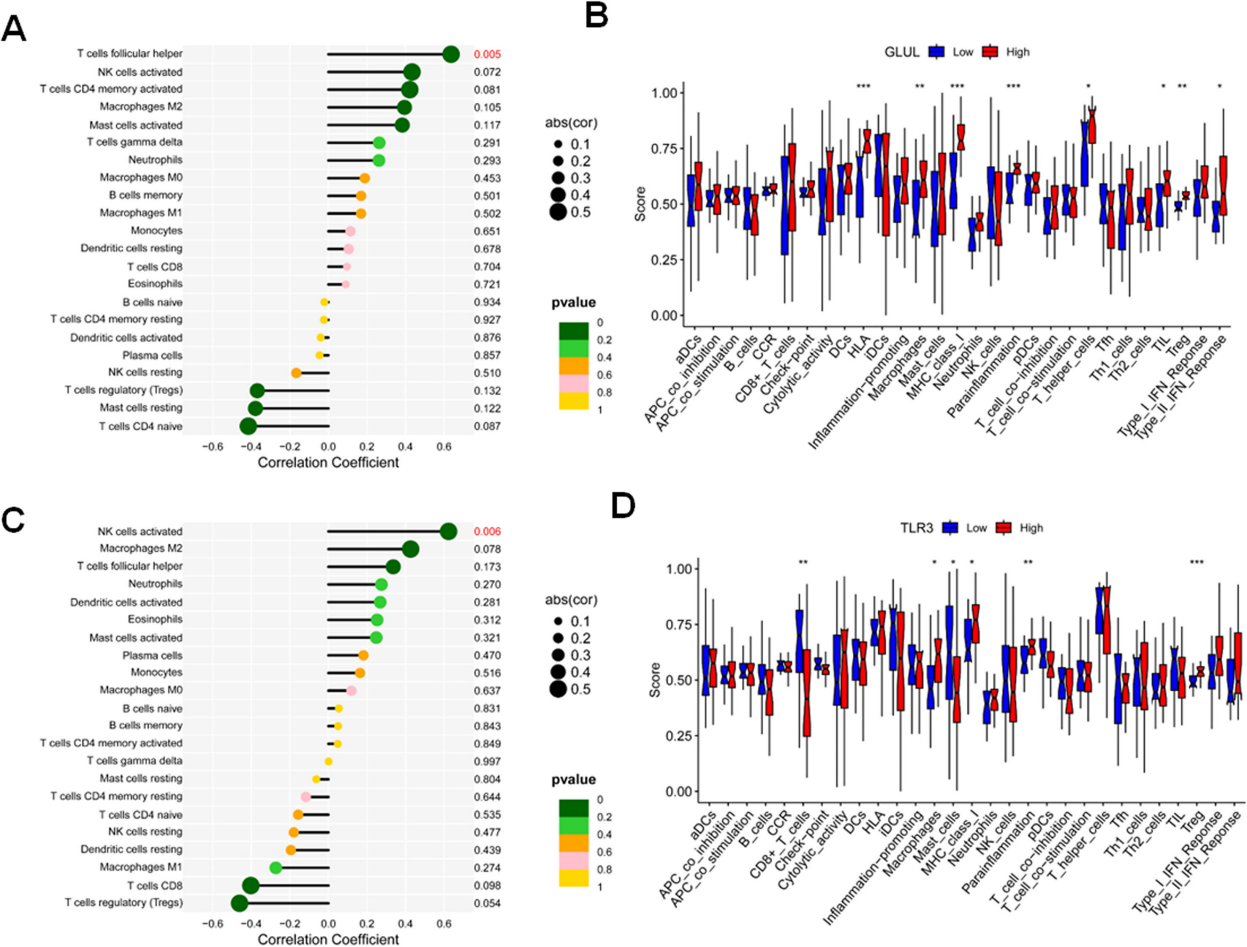
Immune cell infiltration analysis comparing OA patients and normal controls. **(A)** Correlation between GLUL expression and proportions of various immune cell types estimated by CIBERSORT. **(B)** Immune cell infiltration landscape in samples stratified by GLUL expression levels using CIBERSORT. **(C)** Correlation between TLR3 expression and proportions of various immune cell types estimated by CIBERSORT. **(D)** Immune cell infiltration landscape in samples stratified by TLR3 expression levels using CIBERSORT.

To elucidate the immune landscape differences between OA patients and healthy controls, and to explore the association of key genes GLUL and TLR3 with immune cell infiltration, we applied the CIBERSORT algorithm for deconvolution analysis. Correlation analysis revealed that GLUL expression positively correlated with infiltration of follicular helper T cells, activated natural killer (NK) cells, gamma delta T cells, neutrophils, and M2 macrophages. Conversely, GLUL expression was negatively associated with regulatory T cells (Tregs), resting mast cells, and naïve CD4^+^ T cells (**Figure 5A**). Similarly, TLR3 expression positively correlated with neutrophils, activated NK cells, M2 macrophages, and dendritic cells, while showing negative correlations with CD8+ T cells, Tregs, and M1 macrophages (**Figure 5C**). Further, functional enrichment analysis indicated that elevated GLUL expression was linked to immune-related processes including human leukocyte antigen (HLA) expression, macrophage activity, major histocompatibility complex (MHC) class I presentation, parainflammation, T helper cell responses, tumor-infiltrating lymphocytes (TILs), Tregs, and type II interferon responses (**Figure 5B**). In contrast, high TLR3 expression was associated with macrophage function, MHC class I activity, general inflammation, and Treg-related pathways, whereas low TLR3 expression correlated with pathways involving CD8+ T cells and mast cells (**Figure 5D**). These findings highlight distinct immune infiltration patterns associated with GLUL and TLR3 expression, suggesting their potential involvement in modulating the OA immune microenvironment.

### 3.6 Validation and functional characterization of TLR3 in OA

**Figure 6:**
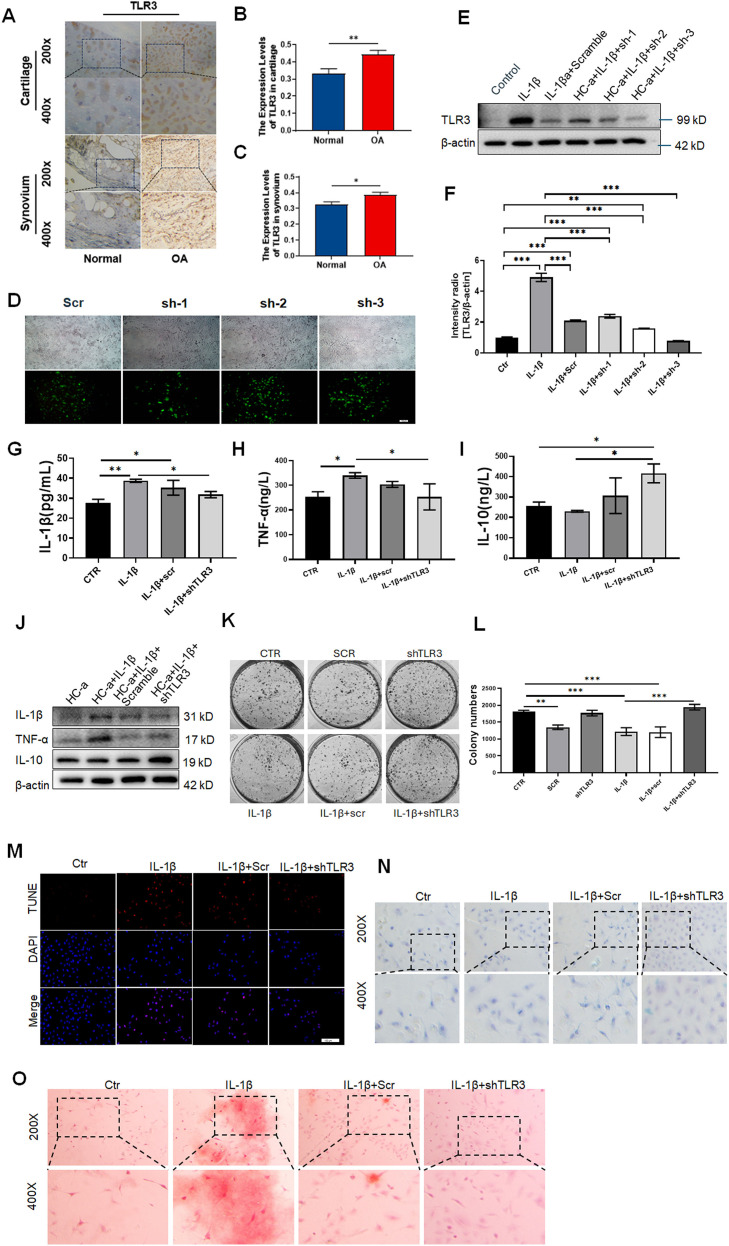
Validation and functional analysis of TLR3 in osteoarthritis. **(A)** Immunohistochemical staining of TLR3 expression in cartilage and synovial tissues from OA patients and healthy controls. **(B)** Quantitative expression of TLR3 in cartilage tissues in **Figure 6A**. **(C)** Quantitative expression of TLR3 in synovial tissues in **Figure 6A**. **(D)** Fluorescence microscopy images showing transduction efficiency of three TLR3-targeting shRNAs in HC-a cells; shRNA-2 exhibited the strongest knockdown effect. **(E)** Western blot analysis confirming reduced TLR3 expression following shRNA-2 transduction in HC-a cells. **(F)** Quantitative expression of intensity ration of TLR3/β-actin. **(G–I)** ELISA results showing the levels of IL-1β, TNF-α, and IL-10 in the supernatant of four experimental groups. **(J)** Western blot analysis of inflammatory cytokine protein levels (IL-1β, TNF-α, IL-10) in the same groups. **(K, L)** Colony formation assays comparing proliferative capacity among control, OA model, and TLR3 knockdown groups. **(M)** TUNEL staining to assess apoptosis in chondrocytes across experimental groups. **(N)** Alcian Blue staining to evaluate acidic mucopolysaccharide content and cartilage matrix integrity. **(O)** Alizarin Red staining to assess calcium deposition and mineralized nodule formation in chondrocytes. Quantitative data are presented as mean ± standard deviation. Statistical comparisons were made using one-way ANOVA followed by *post hoc* tests. *P* < 0.05 was considered statistically significant.

To further elucidate the biological significance of TLR3 in OA, immunohistochemistry was performed on cartilage and synovial tissues obtained from OA patients and healthy donors to validate TLR3 expression *in vivo*, complemented by functional assays using cultured human chondrocytes. Immunohistochemical staining revealed markedly elevated expression of TLR3 in the cartilage and synovial tissues of OA patients relative to healthy controls (**Figures 6A–C**).

Human chondrocyte–derived HC-a cells were selected as a primary *in vitro* model to simulate OA pathology, given their close resemblance to primary articular chondrocytes in phenotype and function. To ensure the reproducibility and cross-species relevance of findings, murine ATDC5 and human immortalized C28/I2 chondrocytic cell lines were also employed in parallel validation experiments. To mimic inflammatory conditions observed in OA joints, HC-a cells were exposed to proinflammatory cytokines, thereby establishing a cellular OA model suitable for functional perturbation studies. To explore the role of TLR3, we designed three shRNA and shRNA-2 exhibited the best transfection and knocking down efficiency (**Figures 6D–F**). Knockdown of TLR3 led to reduced levels of proinflammatory mediators IL-1β and TNF-α, along with an increase in the anti-inflammatory cytokine IL-10 (**Figures 6G–I**). These findings were corroborated by Western blot analysis, which confirmed diminished expression of IL-1β and TNF-α at the protein level (**Figure 6J**). Further phenotypic assessments demonstrated that TLR3 knockdown partially restored cellular proliferation, as indicated by increased colony formation capacity (**Figures 6K, L**). Concurrently, apoptotic activity measured by TUNEL staining was significantly decreased in TLR3-silenced cells compared to the OA model group (**Figure 6M**). To evaluate cartilage matrix integrity, Alcian Blue staining revealed that TLR3 knockdown reversed the loss of acidic mucopolysaccharides observed in the OA model, indicating restoration of the extracellular matrix (**Figure 6N**). Additionally, Alizarin Red staining showed a reduction in calcium deposition and mineralized nodules upon TLR3 silencing, suggesting that aberrant chondrocyte mineralization was attenuated (**Figure 6O**). Collectively, these results suggest that TLR3 inhibition can mitigate key pathological features associated with OA progression.

To assess the reproducibility and generalizability of these findings, all key experiments were independently replicated in two additional chondrocyte cell lines, ATDC5 and C28/I2. The results consistently mirrored those observed in HC-a cells, including similar trends in cytokine expression, cellular proliferation, apoptosis, and extracellular matrix remodeling (**Supplementary Figures S1**, **S2**), thereby confirming the robust proinflammatory and degenerative role of TLR3 in OA pathogenesis. Collectively, these results indicate that TLR3 contributes to the inflammatory response, matrix degradation, and chondrocyte dysfunction associated with OA, underscoring its potential as a pathogenic mediator and therapeutic target.

### 3.7 GLUL overexpression attenuates inflammatory and degenerativephenotypes in chondrocytes

**Figure 7:**
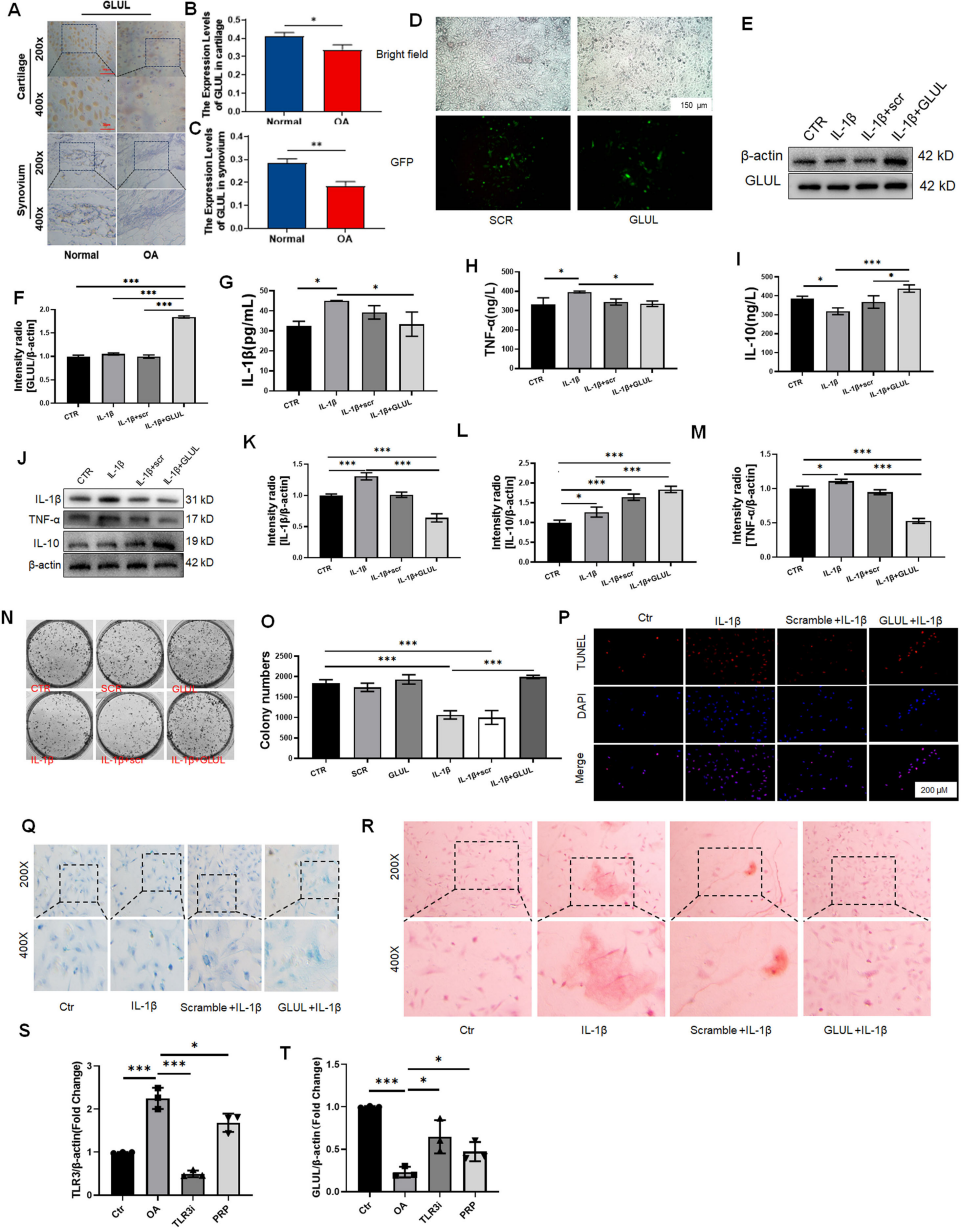
Validation and functional analysis of GLUL in osteoarthritis in patient samples and three cell lines. **(A)** Immunohistochemical staining of GLUL expression in cartilage and synovial tissues from OA patients and healthy controls. **(B)** Quantitative expression of GLUL in cartilage tissues in **Figure 7A**. **(C)** Quantitative expression of GLUL in synovial tissues in **Figure 7A**. **(D)** Fluorescence microscopy images showing transduction efficiency of GLUL-overexpression in HC-a cells. **(E)** Western blot analysis shows the overexpression of GLUL in HC-a cells. **(F)** Quantitative expression of intensity ration of GLUL/β-actin. **(G–I)** ELISA results showing the levels of IL-1β, TNF-α, and IL-10 in the supernatant of four experimental groups. **(J)** Western blot analysis of inflammatory cytokine protein levels (IL-1β, TNF-α, IL-10) in the same groups. **(K, L)** Quantitative analysis of J. **(M–O)** Colony formation assays comparing proliferative capacity among control, OA model, and GLUL overexpression groups. **(P)** TUNEL staining to assess apoptosis in chondrocytes across experimental groups. **(Q)** Alcian Blue staining to evaluate acidic mucopolysaccharide content and cartilage matrix integrity by using HC-a cell line. **(R)** Alizarin Red staining to assess calcium deposition and mineralized nodule formation in chondrocytes by using HC-a cell line. **(S, T)** RT-qPCR analysis of TLR3 and GLUL mRNA in OA-like chondrocytes after TLR3 knockdown (TLR3i) or PRP treatment. **(S)** TLR3 expression is significantly reduced in TLR3i and PRP groups compared to OA controls. **(T)** GLUL expression is significantly increased following TLR3 knockdown and also elevated by PRP treatment, indicating TLR3 negatively regulates GLUL in chondrocytes. Data are mean ± SD; *p < 0.05, p < 0.01 vs OA group.

To elucidate the function of GLUL in OA, we first assessed its expression in patient samples. Immunohistochemical staining revealed a marked reduction of GLUL in both cartilage and synovial tissues from OA patients compared to healthy controls (**Figure 7A**), which was further confirmed by quantitative analysis (**Figures 7B, C**). Next, we overexpressed GLUL in cytokine-induced OA-like HC-a chondrocytes using lentiviral transduction. Transduction efficiency was verified by fluorescence microscopy (**Figure 7D**), and successful overexpression was confirmed by Western blot (**Figure 7E**) and quantification of GLUL/β-actin intensity ratio (**Figure 7F**). GLUL overexpression significantly suppressed inflammatory responses, reduced levels of IL-1β and TNF-α, alongside increased IL-10 in the supernatant (**Figures 7G–I**). Consistent with these results, Western blot analysis of cell lysates demonstrated decreased protein expression of IL-1β and TNF-α, and restoration of IL-10 (**Figure 7J**), which was quantified in **Figures 7K, L**. Overexpression of GLUL also increased the proliferative capacity (**Figures 7M–O**) and reduced cell apoptosis (**Figure 7P**). Alcian Blue staining indicated preservation of acidic mucopolysaccharides and cartilage matrix components (**Figure 7Q**), while Alizarin Red staining showed decreased calcium deposition and mineralized nodule formation, suggesting inhibition of ectopic mineralization (**Figure 7R**).

To validate the generalizability of these effects, all key experiments were independently repeated in two additional chondrocyte lines, ATDC5 and C28/I2. Results were consistent with findings in HC-a cells, demonstrating reproducible anti-inflammatory, anti-apoptotic, and cartilage-preserving effects of GLUL overexpression (**Supplementary Figures S3**, **S4**). To further clarify the hierarchical relationship between TLR3 and GLUL, we performed RT-qPCR analysis in cytokine-induced OA-like chondrocytes. As shown in **Figures 7S, T**, TLR3 knockdown led to a significant upregulation of GLUL mRNA, whereas PRP treatment also increased GLUL while reducing TLR3 expression. These results suggest that TLR3 acts upstream of GLUL, partially mediating its anti-inflammatory and cartilage-protective effects.

Together, these findings suggest that GLUL plays a protective role in OA pathogenesis by modulating inflammatory signaling, preserving chondrocyte viability, and maintaining matrix homeostasis—highlighting its potential as a therapeutic target for OA intervention.

### 3.8 *In vivo* validation of the TLR3 and GLUL and therapeutic intervention in an OA rat model

**Figure 8:**
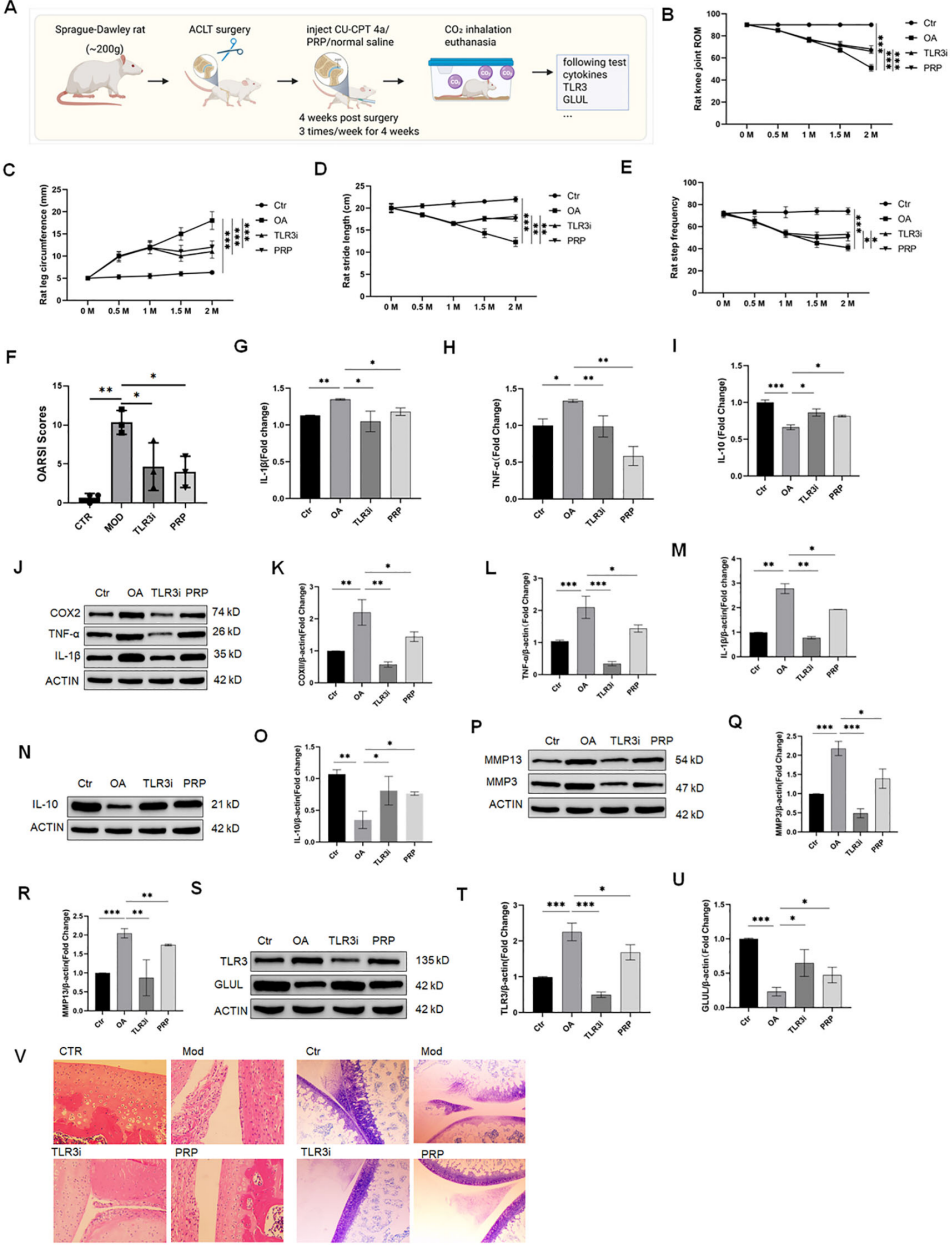
*In vivo* validation of the TLR3/GLUL axis and therapeutic intervention in an OA rat model. **(A)** Schematic illustration of osteoarthritis (OA) induction in Sprague-Dawley rats (~200 g) via anterior cruciate ligament transection (ACLT). Rats were anesthetized using sodium pentobarbital (40–60 mg/kg, i.p.), and a longitudinal incision was made to expose the knee joint. The anterior cruciate ligament was transected, followed by wound closure and postoperative care. OA was established over a 6–8 weeks period. **(B)** Assessment of knee joint range of motion from week 0 to week 8 in sham, model, TLR3 inhibitor, and PRP groups. **(C)** Measurement of knee joint circumference across the 8-week period. **(D)** Quantification of hind limb stride length as a measure of joint mobility. **(E)** Step counts recorded during a 20-second locomotor activity test. **(F)** OARSI scores of knee joints from sham, OA model, TLR3 inhibitor, and PRP-treated groups. OA model group showed significantly higher scores compared with sham controls, indicating cartilage degeneration. Treatment with TLR3 inhibitor or PRP reduced OARSI scores, demonstrating protective effects on cartilage integrity. **(G–I)** Serum cytokine levels assessed by ELISA, including IL-1β **(G)**, TNF-α **(H)**, and IL-10 **(I)**. **(J)** Western blot analysis of inflammatory and anti-inflammatory protein expression in joint tissues. **(K–M)** Protein expression levels of COX2 **(K)**, TNF-α **(M)**, and IL-1β **(M)** in joint tissues. **(O, P)** IL-10 protein expression in joint tissues assessed by Western blot **(O)** and corresponding quantification **(P)**. **(P–R)** Expression of MMP-13 **(Q)** and MMP-3 **(R)** in joint tissues as detected by Western blot. **(S)** Protein expression of TLR3 and GLUL in joint tissues analyzed by Western blot. **(T, U)** Quantitative analysis of TLR3 **(T)** and GLUL **(U)** expression levels. **(V)** Representative immunohistochemistry images of TLR3 expression in knee joint tissues from sham, OA model, TLR3 knockdown, and PRP-treated groups. The images show localization and relative intensity of TLR3 expression in cartilage and synovium. Scale bars: 100 μm. Data are presented as mean ± SD; Statistical significance: *P* < 0.05, P < 0.01, *P* < 0.001; comparisons were made among sham, OA model, TLR3 inhibitor, and PRP-treated groups using one-way ANOVA followed by *post hoc* testing.

To further validate the functional role of the TLR3 and GLUL in OA pathogenesis and evaluate the efficacy of targeted interventions, we established an OA model in Sprague-Dawley rats (~200 g) using anterior cruciate ligament transection (ACLT), a well-established method to induce joint instability and cartilage degeneration (**Figure 8A**). Following model induction, rats were randomly divided into four groups: sham-operated control (n=8), OA model (n=8), TLR3 inhibitor treatment group (CU-CPT 4a, 10 μM, 50 μL; n=7), and platelet-rich plasma (PRP) treatment group (n=7). Intra-articular administration of the respective agents was initiated 4 weeks post-surgery and performed three times per week for a total of 4 weeks. Functional assessments were conducted at baseline and at regular intervals up to 8 weeks post-surgery. These included measurements of knee joint range of motion, joint circumference, stride length, and step count over 20 seconds to evaluate joint mobility and locomotor activity across different groups (**Figures 8B–E**). Cartilage damage was further evaluated using the OARSI scoring system. The OA model group exhibited significantly higher OARSI scores compared to sham controls, confirming successful induction of cartilage degeneration. Treatment with the TLR3 inhibitor or PRP reduced OARSI scores, indicating protective effects on cartilage integrity (**Figure 8F**). To investigate systemic inflammatory responses, serum levels of IL-1β, TNF-α, and IL-10 were measured by ELISA following the treatment period (**Figures 8G–I**). Additionally, local inflammatory status in joint tissues was assessed by Western blot analysis of key pro- and anti-inflammatory proteins, including COX2, TNF-α, IL-1β, and IL-10 (**Figures 8J–O**). Given the critical role of matrix metalloproteinases (MMPs) in cartilage degradation, we further analyzed the expression of MMP-3 and MMP-13 in joint tissues by Western blot (**Figures 8P–R**). Finally, to determine whether the *in vivo* regulatory relationship between TLR3 and GLUL observed *in vitro* was recapitulated in the animal model, we evaluated their protein levels in joint tissues (**Figures 8S–U**). To further investigate the local expression of TLR3 in joint tissues and the effect of TLR3 knockdown or PRP treatment, immunohistochemistry (IHC) staining was performed. Representative images are shown in **Figure 8V**. In sham-operated control joints, TLR3 expression was minimal, primarily localized to the superficial cartilage layer. In contrast, OA model joints exhibited markedly increased TLR3 staining throughout the cartilage and synovial tissues, indicating upregulation associated with OA progression. TLR3 knockdown significantly reduced TLR3 immunoreactivity compared with the OA model, demonstrating effective *in vivo* suppression. PRP-treated joints also showed a moderate decrease in TLR3 expression, consistent with its protective effects on cartilage integrity. These IHC results, together with OARSI scores and Western blot analyses, provide histopathological evidence supporting the *in vivo* regulatory role of TLR3 in OA pathogenesis.

These *in vivo* experiments comprehensively demonstrate the utility of ACLT-induced OA rats for studying TLR3/GLUL signaling and provide a basis for further investigation of TLR3-targeted therapeutic strategies in OA.

## 4 Discussion

**Figure 9:**
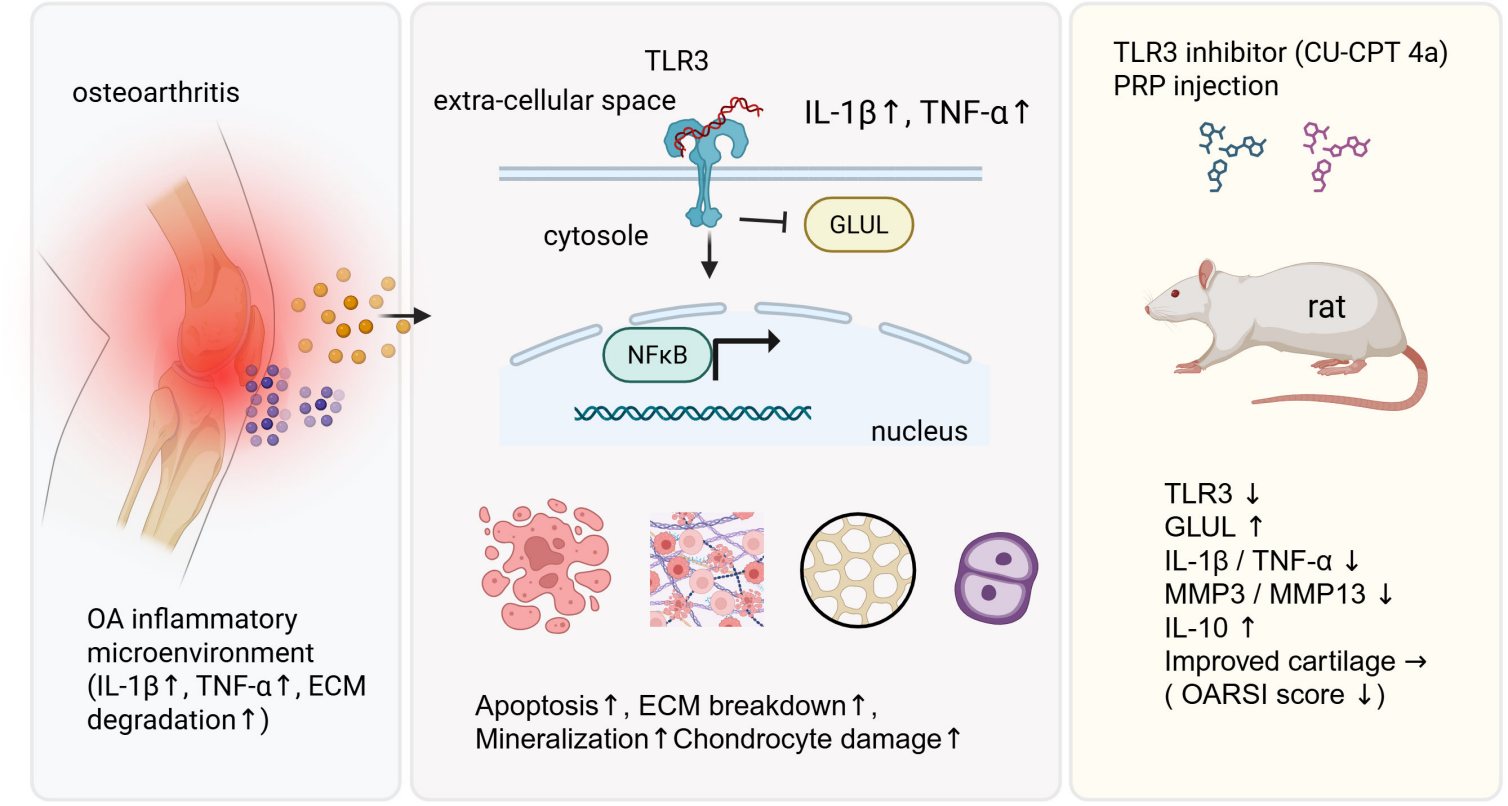
Graphical summary of the TLR3–GLUL axis in osteoarthritis. OA joints exhibit an inflammatory microenvironment characterized by elevated IL-1β, TNF-α, and ECM degradation. TLR3 activation triggers NF-κB signaling, leading to increased inflammatory cytokines, apoptosis, ECM breakdown, mineralization, and chondrocyte damage, accompanied by reduced GLUL. *In vivo* inhibition of TLR3 (CU-CPT4a) combined with PRP injection restores GLUL, suppresses IL-1β/TNF-α and MMP3/MMP13, elevates IL-10, and improves cartilage integrity, reflected by reduced OARSI scores.

In this study, we systematically explored the molecular mechanisms underlying OA by integrating multi-omics analyses with experimental validations. Initial bioinformatics analyses identified NAFRGs that are differentially expressed in OA tissues. Among these, GLUL and TLR3 emerged as key regulators with significant diagnostic potential. Functional enrichment and immune infiltration analyses suggested that these genes influence inflammatory pathways and immune cell dynamics critical to OA progression. Subsequent *in vitro* experiments using human and murine chondrocyte cell lines demonstrated that modulation of TLR3 and GLUL significantly affects inflammatory cytokine production, cell proliferation, apoptosis, and matrix remodeling. Moreover, *in vivo* rat models further validated the pro-inflammatory and cartilage-degenerative roles of TLR3, while GLUL overexpression exhibited protective effects. Collectively, these findings reveal a pivotal TLR3-GLUL axis that orchestrates inflammatory and cell death responses in OA, highlighting novel molecular targets for diagnosis and therapy. Collectively, these findings reveal a pivotal TLR3–GLUL axis that orchestrates inflammatory and cell death responses in OA, highlighting novel molecular targets for diagnosis and therapy(**Figure 9**).

TLR3, a member of the toll-like receptor family, is known to initiate innate immune responses by recognizing double-stranded RNA and activating downstream inflammatory cascades (17, 18). Our data reveal that TLR3 is significantly upregulated in OA cartilage, with elevated expression confirmed in both human OA tissues and *in vitro* models. Mechanistically, TLR3 knockdown led to reduced expression of proinflammatory cytokines, decreased matrix metalloproteinases (MMP3, MMP13), and restoration of cartilage matrix components. Recent study indicates that TLR activation promotes joint degeneration in osteoarthritis (19), TLR3 mediates pro-inflammatory macrophage polarization and chondrocyte hypertrophy in OA, while its inhibition by miR-26b-5p alleviates inflammation and cartilage degeneration (20). Our findings are consistent with the previous research. Functionally, TLR3 silencing reversed the OA-associated phenotypes, including impaired proliferation, increased apoptosis, and excessive mineralization in three distinct chondrocyte lines. Notably, TLR3 may exert its effects via NF-κB or IRF3 signaling pathways, which are known mediators of inflammatory and apoptotic responses in chondrocytes, although the precise signaling cascade in OA requires further investigation. These findings establish TLR3 as a critical upstream modulator of inflammation, cell death, and cartilage degradation in OA.

Conversely, GLUL, encoding glutamine synthetase, plays a fundamental role in glutamine metabolism and redox homeostasis. GLUL promotes M2 polarization and reduces synovial inflammation by modulating the GS/mTORC1 axis in macrophages, offering a potential therapeutic target for OA management (21). Recent studies show that GLUL downregulation promotes macrophage M1 polarization and inflammatory activation, contributing to tissue damage—highlighting a mechanism consistent with its role in osteoarthritis progression (22). We observed a marked downregulation of GLUL in OA tissues, consistent with transcriptomic analyses and histological staining. Overexpression of GLUL in chondrocytes mitigated inflammatory damage by restoring proliferative capacity, reducing apoptosis, normalizing matrix composition, and suppressing abnormal calcification. GLUL metabolites, such as glutamine-derived intermediates, may also influence inflammatory signaling and cell survival, providing an additional layer of regulatory control. Importantly, GLUL overexpression mirrored the protective effects of TLR3 knockdown, suggesting that GLUL may act downstream of TLR3 in regulating cell fate and matrix homeostasis.

Further supporting these findings, *in vivo* studies in an OA rat model confirmed the therapeutic relevance of the TLR3/GLUL axis. TLR3 inhibition significantly alleviated OA-associated inflammation, as evidenced by decreased serum levels of IL-1β and TNF-α and restored IL-10 expression. Western blot analysis of joint tissues revealed a concomitant decrease in COX2, MMPs, and proinflammatory cytokines, along with an increase in GLUL protein levels. Similar trends were observed following PRP treatment, which served as a positive control. It should be noted that PRP contains multiple bioactive components, and its protective effects on OA cartilage may be partially mediated through modulation of the TLR3/GLUL axis, although other mechanisms are also likely involved. These *in vivo* results strongly validate the *in vitro* findings and implicate the TLR3–GLUL axis as a key regulator of OA progression. Integrative pathway analyses provided further insights into the mechanisms by which these genes modulate OA. GSEA and GSVA revealed that TLR3 is associated with pathways involved in ubiquitin-mediated proteolysis and neurodegenerative disorders, whereas GLUL is implicated in oxidative phosphorylation and redox balance. Immune infiltration analyses indicated that TLR3 expression correlates with proinflammatory immune subsets such as M2 macrophages and dendritic cells, while GLUL expression positively correlates with anti-inflammatory and immune-regulating cells including Tfh and activated NK cells. Notably, the CIBERSORT algorithm estimates immune cell proportions via computational deconvolution based on reference gene expression profiles. This method provides an indirect inference of immune composition and may not fully reflect the actual immune landscape within complex joint tissues such as cartilage and synovium. The accuracy of the deconvolution inherently depends on the quality and relevance of the reference dataset, which was originally derived from peripheral blood leukocytes. Therefore, the immune infiltration patterns observed in this study should be interpreted as estimates rather than precise quantifications. Future validation of key immune cell subsets, particularly M2 macrophages, in clinical OA samples using immunohistochemistry or flow cytometry would further strengthen the robustness of these findings. Together, these results suggest that TLR3 exacerbates OA by promoting inflammation and ferroptosis, whereas GLUL exerts a protective role by restoring metabolic homeostasis and limiting chondrocyte damage.

Study Limitations: Several limitations should be noted. First, bioinformatics analyses are inherently predictive and require further experimental validation. Second, immortalized chondrocyte cell lines used *in vitro* may not fully recapitulate primary chondrocyte biology. Third, differences between rat OA models and human disease may affect translatability. Finally, the precise molecular mechanisms linking TLR3 and GLUL remain to be elucidated, including potential post-transcriptional or signaling-mediated regulation.

This study is among the first to experimentally validate the functional interplay between TLR3 and GLUL in OA across transcriptomic, cellular, and animal levels. Targeting this axis offers a promising strategy to simultaneously regulate inflammatory signaling, cell death, and matrix degradation in OA. Nevertheless, future research should aim to delineate the precise molecular interactions between TLR3 and GLUL, including whether GLUL is transcriptionally or post-transcriptionally regulated by TLR3-dependent pathways. Future studies should aim to delineate the detailed signaling pathways mediating TLR3 and GLUL interactions.

## 5 Conclusion

In summary, our study reveals TLR3 and GLUL as key modulators that integrate immune responses and regulated cell death pathways in OA. TLR3 amplifies inflammatory cascades and catabolic processes, whereas GLUL contributes to cellular homeostasis through metabolic and anti-inflammatory functions. Using integrated transcriptomic analyses, *in vitro* functional assays, and *in vivo* validation, we provide comprehensive evidence that modulating the TLR3 and GLUL can effectively alleviate inflammation, suppress apoptosis, and mitigate cartilage degeneration in OA. These findings advance the current understanding of OA pathogenesis and support the potential of this axis as a promising target for therapeutic development.

## Data Availability

The original contributions presented in the study are included in the article/**Supplementary Material**. Further inquiries can be directed to the corresponding author.

## Supplementary material

Supplementary Figure 1 Validation and functional analysis of TLR3 in osteoarthritis by using C28/12 cell line. **(A)** Fluorescence microscopy images show transduction efficiency of three TLR3-targeting shRNAs in C28/12 cells; shRNA-2 exhibited the strongest knockdown effect. **(B)** Western blot analysis confirming reduced TLR3 expression following shRNA-2 transduction in C28/12 cells. **(C)** Quantitative expression of intensity ration of TLR3/β-actin. **(D–F)** ELISA results showing the levels of IL-1β, TNF-α, and IL-10 in the supernatant of four experimental groups.**(G)** Western blot analysis of inflammatory cytokine protein levels (IL-1β, TNF-α, IL-10) in the same groups. **(H–J)** The intensity ration between IL-1β/β-actin, IL-10/β-actin, TNF-α/β-actin after knocking down of TLR3. **(K)** Colony formation assays comparing proliferative capacity among control, OA model, and TLR3 knockdown groups. **(L)** Quantitative analysis of K. **(M)** TUNEL staining to assess apoptosis in chondrocytes across experimental groups in C28/12 cells. **(N)** Alcian Blue staining to evaluate acidic mucopolysaccharide content and cartilage matrix integrity with C28/12 cells. **(O)** Alizarin Red staining to assess calcium deposition and mineralized nodule formation in chondrocytes. *P < 0.05; **P < 0.01; ***P < 0.001.

Supplementary Figure 2 Validation and functional analysis of TLR3 in osteoarthritis by using ATDC5 cell line. **(A)** Fluorescence microscopy images show transduction efficiency of three TLR3-targeting shRNAs in ATDC5 cells; shRNA-2 exhibited the strongest knockdown effect. **(B)** Western blot analysis confirming reduced TLR3 expression following shRNA-2 transduction in ATDC5 cells. **(C)** Quantitative expression of intensity ration of TLR3/β-actin. **(D–F)** ELISA results showing the levels of IL-1β, TNF-α, and IL-10 in the supernatant of four experimental groups. **(G)** Western blot analysis of inflammatory cytokine protein levels (IL-1β, TNF-α, IL-10) in the same groups. **(H–J)** The intensity ration between IL-1β/β-actin, IL-10/β-actin, TNF-α/β-actin after knocking down of TLR3. **(K)** Colony formation assays comparing proliferative capacity among control, OA model, and TLR3 knockdown groups. **(L)** Quantitative analysis of K. **(M)** TUNEL staining to assess apoptosis in chondrocytes across experimental groups in ATDC5 cells. **(N)** Alcian Blue staining to evaluate acidic mucopolysaccharide content and cartilage matrix integrity with ATDC5 cells. **(O)** Alizarin Red staining to assess calcium deposition and mineralized nodule formation in chondrocytes. *P < 0.05; **P < 0.01; ***P < 0.001.

Supplementary Figure 3 Validation and functional analysis of GLUL in osteoarthritis by using C28/12 cell line. **(A)** Fluorescence microscopy images showS transduction efficiency of GLUL overexpression in C28/12 cells. **(B)** Western blot analysis shows the overexpression of GLUL in C28/12 cells. **(C)** Quantitative expression of intensity ration of GLUL/β-actin. **(D–F)** ELISA results showing the levels of IL-1β, TNF-α, and IL-10 in the supernatant of four experimental groups. **(G)** Western blot analysis of inflammatory cytokine protein levels (IL-1β, TNF-α, IL-10) after the overexpression of GLUL in C28/12 cells. **(H–J)** The intensity ration between IL-1β/β-actin, IL-10/β-actin, TNF-α/β-actin after the overexpression of GLUL in C28/12 cells. **(K)** Colony formation assays comparing proliferative capacity among control, OA model, and GLUL overexpression groups. **(L)** Quantitative analysis of K. **(M)** TUNEL staining to assess apoptosis in chondrocytes across experimental groups in C28/12 cells. **(N)** Alcian Blue staining to evaluate acidic mucopolysaccharide content and cartilage matrix integrity with C28/12 cells. **(O)** Alizarin Red staining to assess calcium deposition and mineralized nodule formation in chondrocytes. *P < 0.05; **P < 0.01; ***P < 0.001.

Supplementary Figure 4 Validation and functional analysis of GLUL in osteoarthritis by using ATDC5 cell line. **(A)** Fluorescence microscopy images show transduction efficiency of GLUL overexpression in ATDC5 cells. **(B)** Western blot shows the GLUL expression following overexpression of GLUL in ATDC5 cells. **(C)** Quantitative expression of intensity ration of GLUL/β-actin. **(D–F)** ELISA results showing the levels of IL-1β, TNF-α, and IL-10 in the supernatant of four experimental groups. **(G)** Western blot analysis of inflammatory cytokine protein levels (IL-1β, TNF-α, IL-10) in the same groups. **(H–J)** The intensity ration between IL-1β/β-actin, IL-10/β-actin, TNF-α/β-actin after overexpression of GLUL. **(K)** Colony formation assays comparing proliferative capacity among control, OA model, and GLUL overexpression groups. **(L)** Quantitative analysis of K. **(M)** TUNEL staining shows cell apoptosis in chondrocytes across experimental groups in ATDC5 cells. **(N)** Alcian Blue staining shows acidic mucopolysaccharide content and cartilage matrix integrity with ATDC5 cells. **(O)** Alizarin Red staining to assess calcium deposition and mineralized nodule formation in chondrocytes. *P < 0.05; **P < 0.01; ***P < 0.001.
