# Supplementary figures and images for: TLR3 and GLUL orchestrate inflammatory and homeostatic imbalance in osteoarthritis
Source: Front Immunol. 2026 Jan 23;16:1650375. doi: 10.3389/fimmu.2025.1650375 (PMC12875911; doi:10.3389/fimmu.2025.1650375)

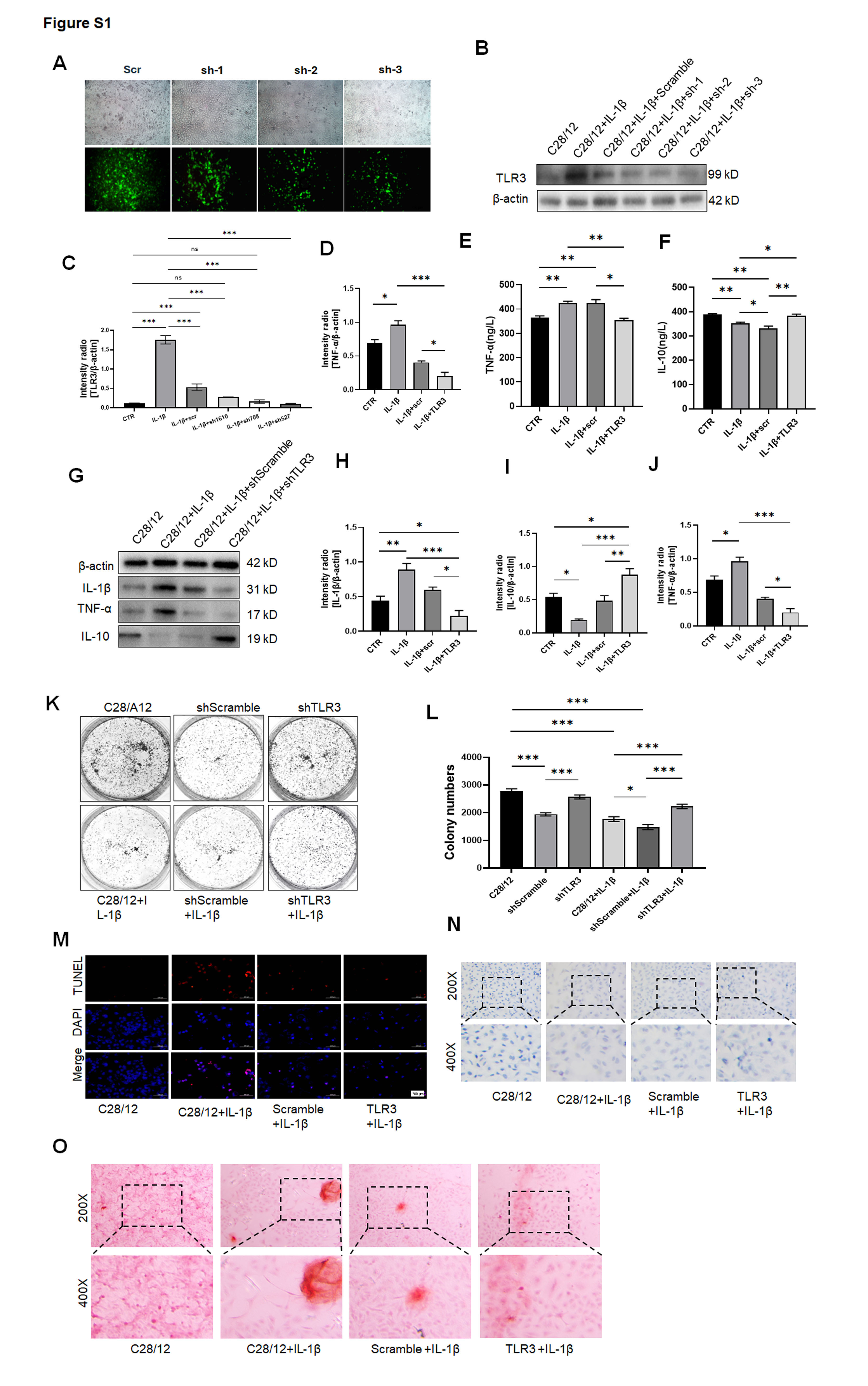

Supplement: Supplementary Figure 1 — Validation and functional analysis of TLR3 in osteoarthritis by using C28/12 cell line. (A) Fluorescence microscopy images show transduction efficiency of three TLR3-targeting shRNAs in C28/12 cells; shRNA-2 exhibited the strongest knockdown effect. (B) Western blot analysis confirming reduced TLR3 expression following shRNA-2 transduction in C28/12 cells. (C) Quantitative expression of intensity ration of TLR3/β-actin. (D–F) ELISA results showing the levels of IL-1β, TNF-α, and IL-10 in the supernatant of four experimental groups.(G) Western blot analysis of inflammatory cytokine protein levels (IL-1β, TNF-α, IL-10) in the same groups. (H–J) The intensity ration between IL-1β/β-actin, IL-10/β-actin, TNF-α/β-actin after knocking down of TLR3. (K) Colony formation assays comparing proliferative capacity among control, OA model, and TLR3 knockdown groups. (L) Quantitative analysis of K. (M) TUNEL staining to assess apoptosis in chondrocytes across experimental groups in C28/12 cells. (N) Alcian Blue staining to evaluate acidic mucopolysaccharide content and cartilage matrix integrity with C28/12 cells. (O) Alizarin Red staining to assess calcium deposition and mineralized nodule formation in chondrocytes. *P < 0.05; **P < 0.01; ***P < 0.001. [file Image1.jpeg]

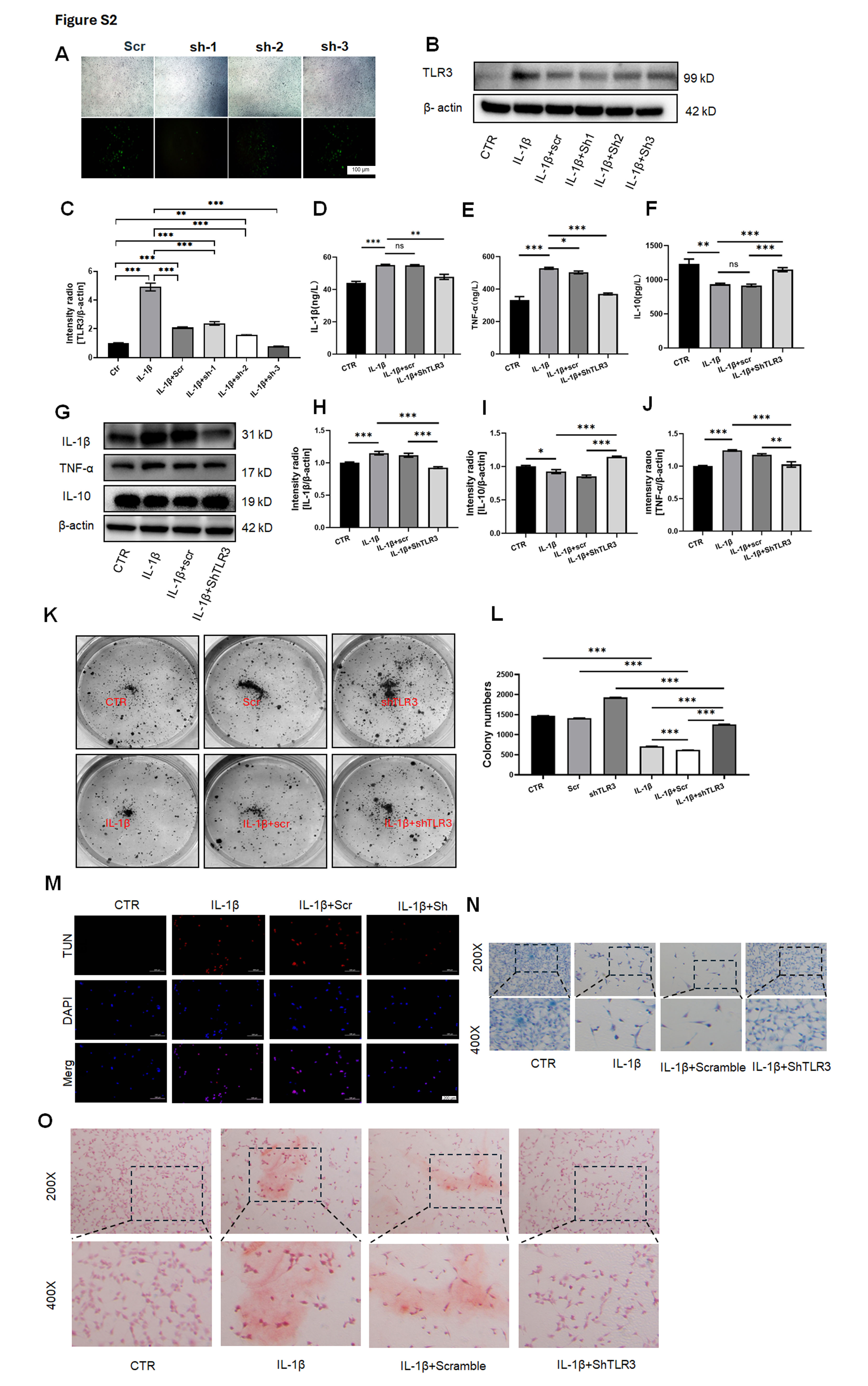

Supplement: Supplementary Figure 2 — Validation and functional analysis of TLR3 in osteoarthritis by using ATDC5 cell line. (A) Fluorescence microscopy images show transduction efficiency of three TLR3-targeting shRNAs in ATDC5 cells; shRNA-2 exhibited the strongest knockdown effect. (B) Western blot analysis confirming reduced TLR3 expression following shRNA-2 transduction in ATDC5 cells. (C) Quantitative expression of intensity ration of TLR3/β-actin. (D–F) ELISA results showing the levels of IL-1β, TNF-α, and IL-10 in the supernatant of four experimental groups. (G) Western blot analysis of inflammatory cytokine protein levels (IL-1β, TNF-α, IL-10) in the same groups. (H–J) The intensity ration between IL-1β/β-actin, IL-10/β-actin, TNF-α/β-actin after knocking down of TLR3. (K) Colony formation assays comparing proliferative capacity among control, OA model, and TLR3 knockdown groups. (L) Quantitative analysis of K. (M) TUNEL staining to assess apoptosis in chondrocytes across experimental groups in ATDC5 cells. (N) Alcian Blue staining to evaluate acidic mucopolysaccharide content and cartilage matrix integrity with ATDC5 cells. (O) Alizarin Red staining to assess calcium deposition and mineralized nodule formation in chondrocytes. *P < 0.05; **P < 0.01; ***P < 0.001. [file Image2.jpeg]

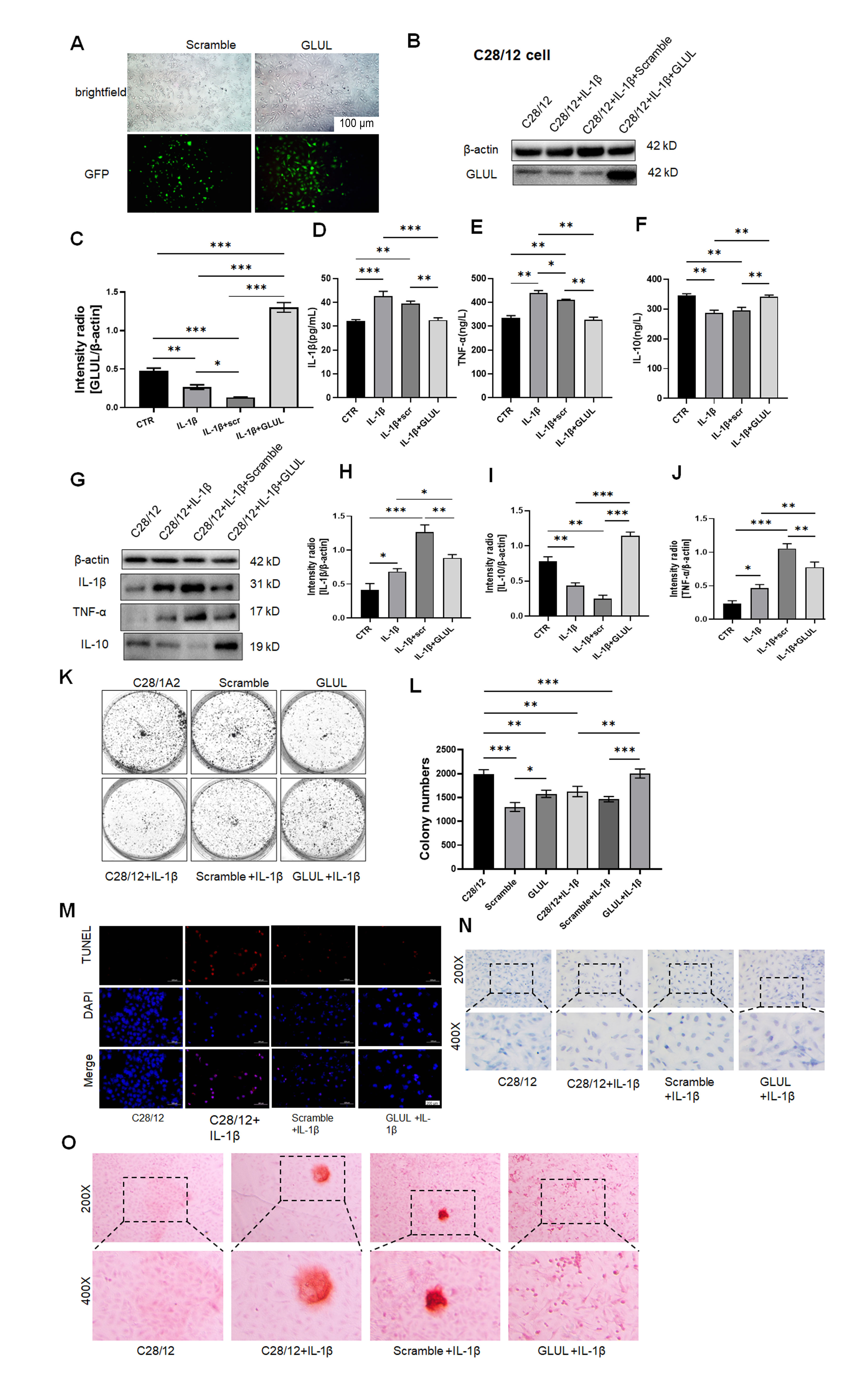

Supplement: Supplementary Figure 3 — Validation and functional analysis of GLUL in osteoarthritis by using C28/12 cell line. (A) Fluorescence microscopy images showS transduction efficiency of GLUL overexpression in C28/12 cells. (B) Western blot analysis shows the overexpression of GLUL in C28/12 cells. (C) Quantitative expression of intensity ration of GLUL/β-actin. (D–F) ELISA results showing the levels of IL-1β, TNF-α, and IL-10 in the supernatant of four experimental groups. (G) Western blot analysis of inflammatory cytokine protein levels (IL-1β, TNF-α, IL-10) after the overexpression of GLUL in C28/12 cells. (H–J) The intensity ration between IL-1β/β-actin, IL-10/β-actin, TNF-α/β-actin after the overexpression of GLUL in C28/12 cells. (K) Colony formation assays comparing proliferative capacity among control, OA model, and GLUL overexpression groups. (L) Quantitative analysis of K. (M) TUNEL staining to assess apoptosis in chondrocytes across experimental groups in C28/12 cells. (N) Alcian Blue staining to evaluate acidic mucopolysaccharide content and cartilage matrix integrity with C28/12 cells. (O) Alizarin Red staining to assess calcium deposition and mineralized nodule formation in chondrocytes. *P < 0.05; **P < 0.01; ***P < 0.001. [file Image3.jpeg]

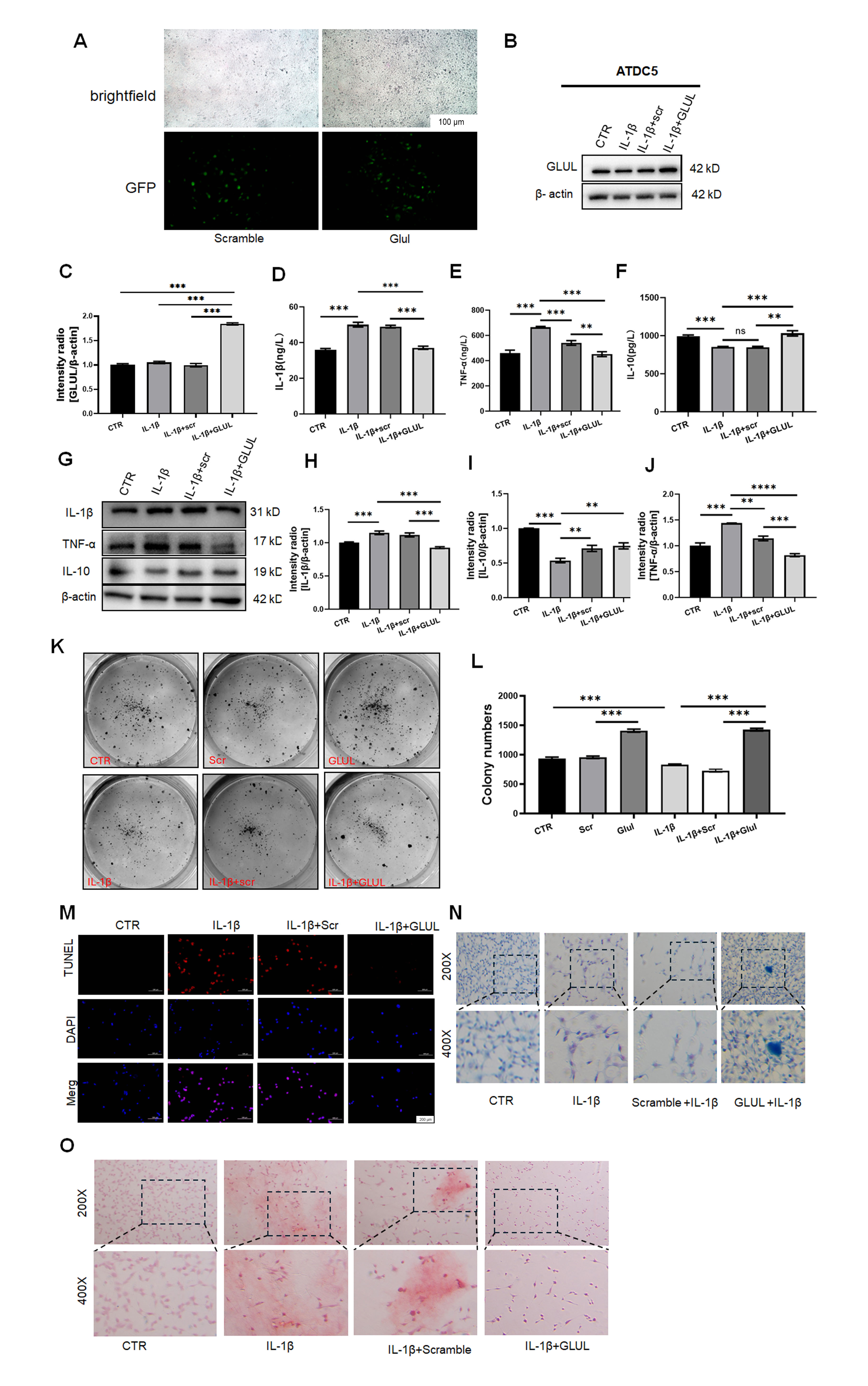

Supplement: Supplementary Figure 4 — Validation and functional analysis of GLUL in osteoarthritis by using ATDC5 cell line. (A) Fluorescence microscopy images show transduction efficiency of GLUL overexpression in ATDC5 cells. (B) Western blot shows the GLUL expression following overexpression of GLUL in ATDC5 cells. (C) Quantitative expression of intensity ration of GLUL/β-actin. (D–F) ELISA results showing the levels of IL-1β, TNF-α, and IL-10 in the supernatant of four experimental groups. (G) Western blot analysis of inflammatory cytokine protein levels (IL-1β, TNF-α, IL-10) in the same groups. (H–J) The intensity ration between IL-1β/β-actin, IL-10/β-actin, TNF-α/β-actin after overexpression of GLUL. (K) Colony formation assays comparing proliferative capacity among control, OA model, and GLUL overexpression groups. (L) Quantitative analysis of K. (M) TUNEL staining shows cell apoptosis in chondrocytes across experimental groups in ATDC5 cells. (N) Alcian Blue staining shows acidic mucopolysaccharide content and cartilage matrix integrity with ATDC5 cells. (O) Alizarin Red staining to assess calcium deposition and mineralized nodule formation in chondrocytes. *P < 0.05; **P < 0.01; ***P < 0.001. [file Image4.jpeg]
